# Supplementary material for: General-purpose programmable photonic processor for advanced radiofrequency applications
Source: Nat Commun. 2024 Feb 20;15:1563. doi: 10.1038/s41467-024-45888-7 (PMC10879507; doi:10.1038/s41467-024-45888-7)
Supplement: Supplementary file 1 — Supplementary Information [file 41467_2024_45888_MOESM1_ESM.pdf]

Supplementary notes for:

General-purpose programmable photonic processor for advanced radiofrequency applications

Daniel Pérez-López[1,2], Ana Gutierrez[1,2], David Sánchez[2], Aitor López[1], Mikel Gutierrez[2], Erica Sánchez[1,2], Juan Fernández[2], Alejandro Cruz[2], Alberto Quirós[2], Zhenyun Xie[2], Jesús Benitez[2], Nandor Bekesi[2], Alejandro Santomé[2], Diego Pérez-Galacho[1], Prometheus DasMahapatra[1] Andrés Macho[1] and José Capmany[1,2]

[1]*Photonics Research Labs, iTEAM Research Institute, Universitat Politècnica de València, Valencia, Spain*

[2]*iPronics, Programmable Photonics, Valencia, Spain*

Supplementary Note 1: Description and operation of a microwave photonic system

Supplementary Note 2: The programmable processor

Supplementary Note 3: Loss and true time delay characterization of the processor photonic core

Supplementary Note 4: Implementation of tunable and reconfigurable optical filters

Supplementary Note 5: Implementation of tunable and reconfigurable RF filters and phase shifters

Supplementary Note 6: Optical generation of MM-wave CW signals.

Supplementary Note 7: Arbitrary RF waveform generation.

Supplementary Note 8: Tuneable delay lines and beamforming.

Supplementary Note 9: 5G signal interconnection and power division.

Supplementary Note 10: Switching, broadcasting and add-drop multiplexing.

Supplementary Note 11: Tunable frequency measurement

Supplementary Note 12: Implementation of Optoelectronic Oscillators

Supplementary Note 1

Description and operation of a microwave photonic system

Supplementary Fig.1 shows the layouts of a classical RF and of a RF-Photonics System. In the classical configuration depicted in Supplementary Fig. 1(a), the RF signal originating from a generator or detected by an antenna or an antenna array is directly processed in the RF domain (f_{RF}) by RF components before being detected or re-radiated. In the RF-Photonic system shown in Supplementary Fig. 1(b), the RF signal is first upconverted to the optical domain (f_o+f_{RF}) using an external modulator fed by a continuous wave laser source emitting at the optical frequency f_o. It is then processed in the optical domain by photonic reconfigurable components featuring broadband operation and lower losses, before being down-converted back to the RF domain.

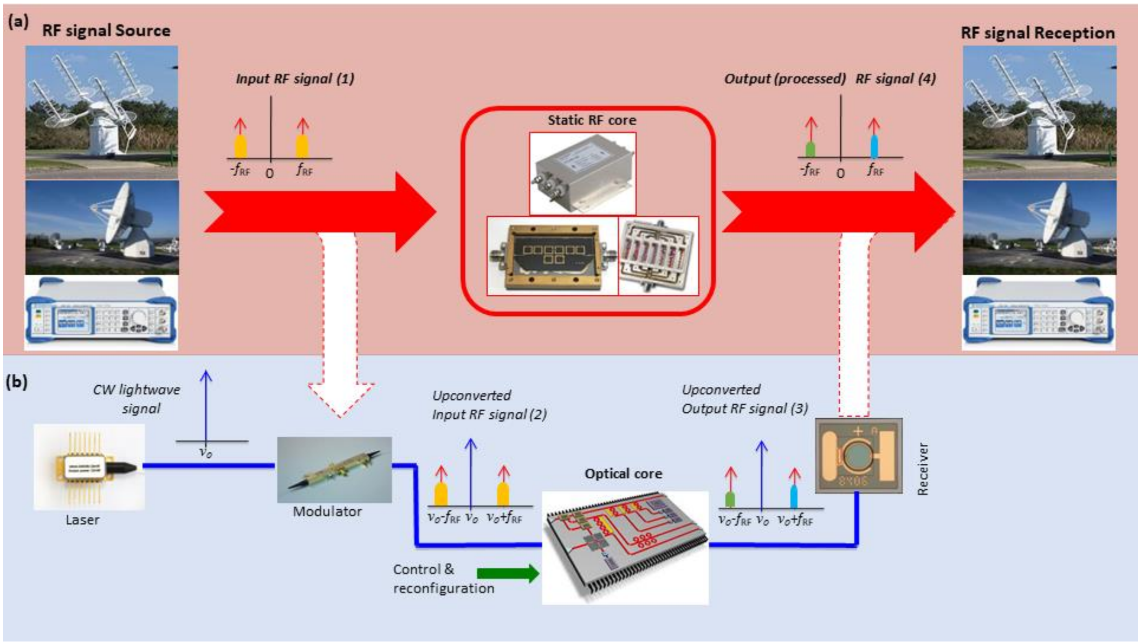

Supplementary Fig. 1: **Layouts of RF systems** (a) Classical RF system , (b) RF-Photonics system.

Supplementary Note 2.

The programmable processor

Programmable photonics involves a software framework shown in Supplementary Fig. 2 that encompasses a wide array of algorithms, methods, or routines executed by an electronic processing unit. These serve primarily to configure specific functionalities within a Photonic Integrated Circuit (PIC) and to optimize the backplane.

These routines can be categorized based on their intended outcomes or their specific requirements. Among them, a group of fundamental routines is dedicated to calibrating and pre-characterizing the photonic integrated circuit. For instance, iterative routines can be employed to calibrate the electro-optical response of each phase shifter within the circuit, eliminating the need for integrating optoelectronic monitors into the waveguide mesh arrangement[1]. Additionally, these routines can extract valuable information such as the power consumption of individual units, accumulated losses, and identifying and recording defects within the circuit.

The data collected through these routines can then be utilized by a set of algorithms designed to optimize both the circuit's functionality and resource utilization. One such algorithm is the auto-routing algorithm[2].

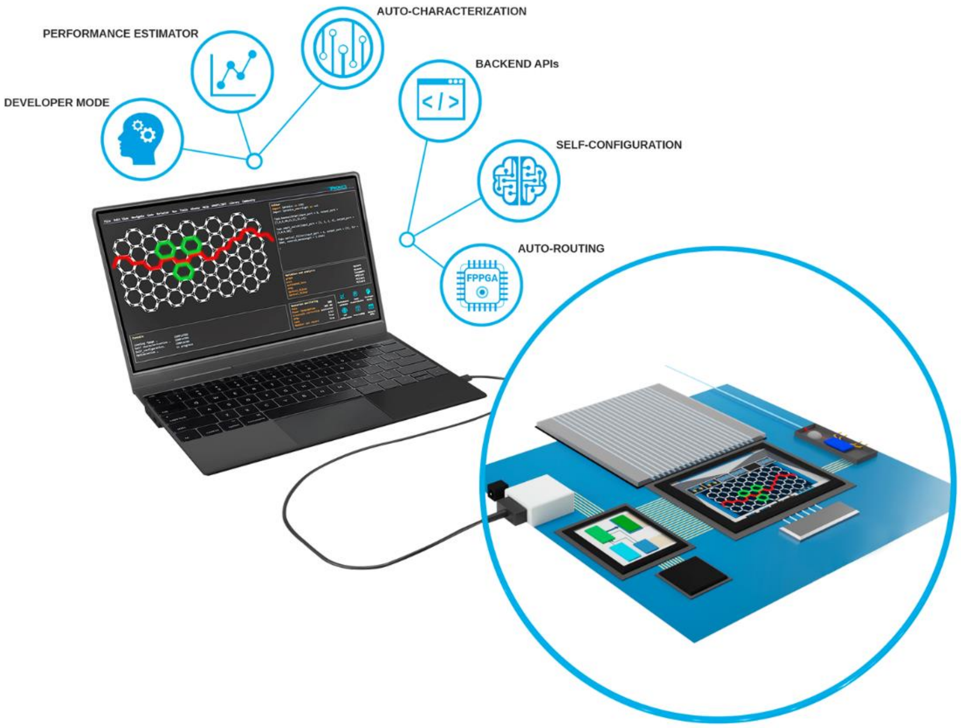

Supplementary Fig. 2: **Artist view of the photonic processor including the full technology stack** with control electronics and software layer including backends, simulation tools, and automated libraries.

Graph-based modelling

In the realm of waveguide mesh-based circuits, a critical aspect is the configuration of optical interconnections and delay lines. This text also highlights the potential of Field-Programmable Photonic Gate Arrays (FPPGAs) to interface with advanced components within their core.

In essence, the future development of large-scale programmable Photonic Integrated Circuits (PICs) hinges on the maturation of several key enabling technologies. These include the imperative need for low loss, low power consumption, and compact unit cells, as well as dense electrical interfacing for low-speed signals and the co-integration of high-speed electronics. Simultaneously, advancements in the capabilities of the software layer will evolve to alleviate the reliance on perfect components, enabling the creation of robust and fault-tolerant circuits.

In both of these technological aspects, the pivotal role played by the configuration of optical connections and optical delay lines cannot be overstated. It involves selecting and arranging programmable unit cells in a manner that defines the path taken by the optical signal. For a given specification, such as achieving a 10 ns delay between two specific optical ports, multiple solutions may exist. In this context, the development of a software routine capable of determining the required drive power and automatically controlling the optimal path concerning factors like loss, power consumption, and other non-ideal effects is a must.

Once the translation of waveguide meshes into a graph mapping the nodes and internal connections of the Programmable Unit Cell (PUC) is achieved, graph theory and modified pathfinding algorithms come into play. These techniques facilitate the search for the optimal circuit configuration, as evidenced by references[2,3]. The resultant configurations can be stored for future use and employed to dynamically configure the circuit.

The auto-routing algorithm assumes a critical role in this process, as it automatically determines optical paths and interferometric structures based on the waveguide mesh topology and a set of specifications. Furthermore, this routine introduces groundbreaking self-healing and fault-tolerant capabilities for the PIC. When confronted with damaged areas within the circuit, the algorithm is adept at identifying alternative sub-optimal paths through the photonic arrangement.

Configuration automation in programmable photonic circuits

Computational optimization and machine learning techniques have emerged as valuable tools across various application domains. Specifically, they have found utility in configuring optical filters and multiport interferometers, streamlining complex tasks. Notably, these techniques have typically been applied to singular applications.

Recent advancements have ventured into the formidable challenge of configuring waveguide mesh arrangements using advanced optimization methods. In this context, the phase shifters assume the role of adjustable parameters in an optimization endeavor. Methods such as gradient descent and non-derivative approaches prove instrumental in minimizing a cost function tailored to the specific application at hand[2,4]. Remarkably, these methods automatically account for non-ideal effects, encompassing both dynamic and passive factors, as integral components of the system's behavior.

Circuit emulation and simulation of hyper-coupled structures

The emergence of multipurpose programmable photonic processors addresses the need for cost-effective and versatile solutions across a wide range of photonic applications.

Extending the optical core opens up possibilities for creating more intricate structures and functionalities. However, this expansion introduces design trade-offs, as it brings about parasitic effects stemming from the fabrication process and dynamic operation of the devices.

Designing and validating such complex systems, both in hardware and software, typically involve expensive, risky, and time-consuming processes. To address these challenges, some works introduce and compare simulation tools designed to predict the spectral response of any 2D integrated photonic mesh circuit, regardless of the number of coupled cells it comprises[5]. These simulation methods not only reduce development costs but also accelerate the growth of new circuit designs. They stand as essential tools for the advancement of programmable photonic libraries, streamlining the development of these cutting-edge technologies.

Supplementary Note 3.

Loss and true time delay characterization of the processor photonic core

Before the synthesis of any photonic integrated circuit, we characterized each PUC in the photonic waveguide mesh and, in particular, their losses. The overall insertion loss of the PUCs can be obtained by measuring different interconnection links in the mesh, that is, paths traversing a different number of PUCs. For such purpose, we used the basic optical setup shown in Supplementary Fig. 3, which consists of a tunable laser source (TLS), ANDO AQ4321D, a polarization controller to control the polarization of the injected light to the chip, and an optical spectra analyzer (OSA), ANDO AQ6317C, to capture the spectra of each synthesized interconnect.

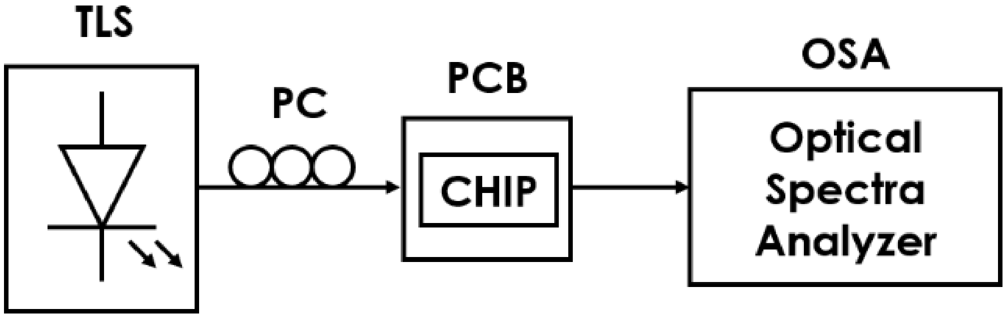

Supplementary Fig. 3: **Passive setup used to measure the spectra of the different optical interconnects in the processor waveguide mesh**.

To avoid results deviations due to human connection errors in the lab, we used the same input and output ports for interconnects of different lengths. We carried out each experiment twice, first for paths traversing an odd number of PUCs and second, for paths traversing an even number of PUCs. Moreover, we synthesized interconnects traversing an equal number of PUCs to check the dispersion in the measured results. For interconnects traversing an odd number of PUCs, we used input port 0 and output port 6 as shown in Supplementary Fig. 4(b) where we present the spectra of the measured interconnects within a 20nm wavelength range and for up to 7 interconnects of each length. For each interconnect length the spectra shown are the mean and the error bars represent the standard deviation.

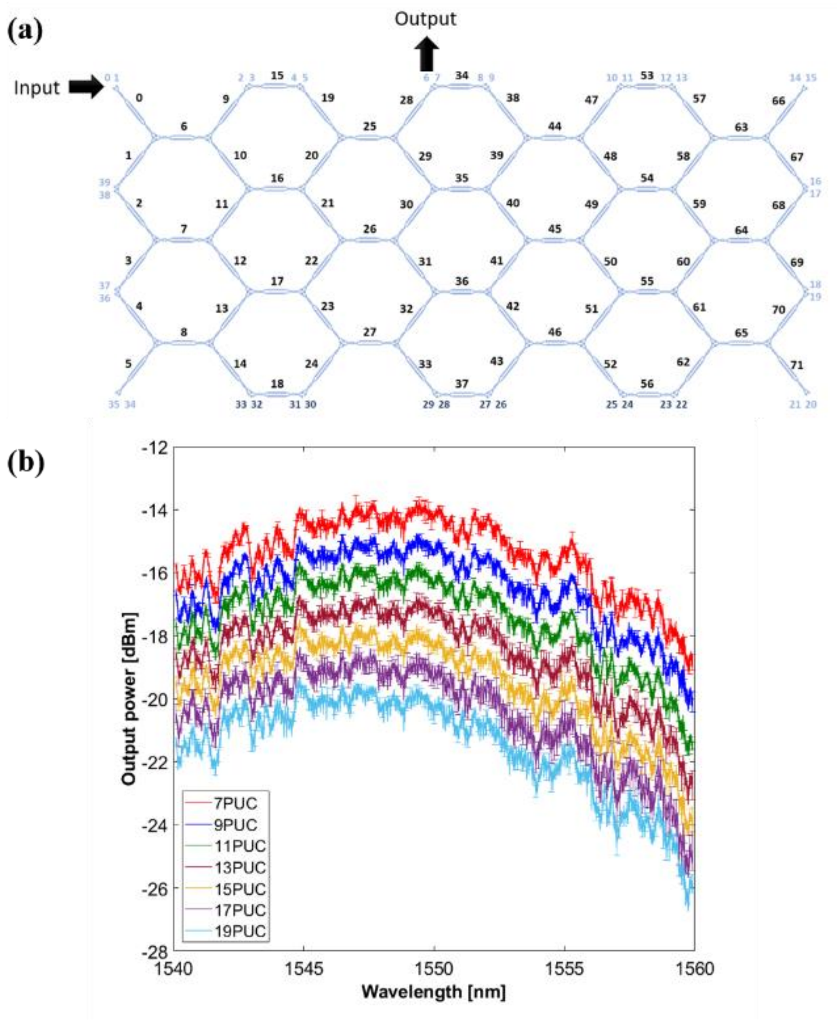

Supplementary Fig. 4: **Illustration of the cutback method for waveguide loss measurement in the waveguide mesh (odd number of connections).** (a) Input 0 and Output 6 ports used for the measured interconnect of different lengths and (b) Mean spectra and standard deviation.

From the measured spectra, we obtained the PUC losses at 1550nm using the cutback method. In Supplementary Fig. 5 (a) the fitting for all the measured interconnects is shown, while in Supplementary Fig. 5(b) and Supplementary Fig. 5 (c) the fitting is made for the maxima and mean spectrum respectively. In all cases, the obtained PUC losses are around 0.45dB/PUC and the excess losses around 10.5dB, which include the coupling losses and the propagation and bend losses encountered in the path through the mesh (crossing the monitoring unit array). Moreover, from Supplementary Fig. 5 (a), a low dispersion between interconnects of the same lengths is observed.

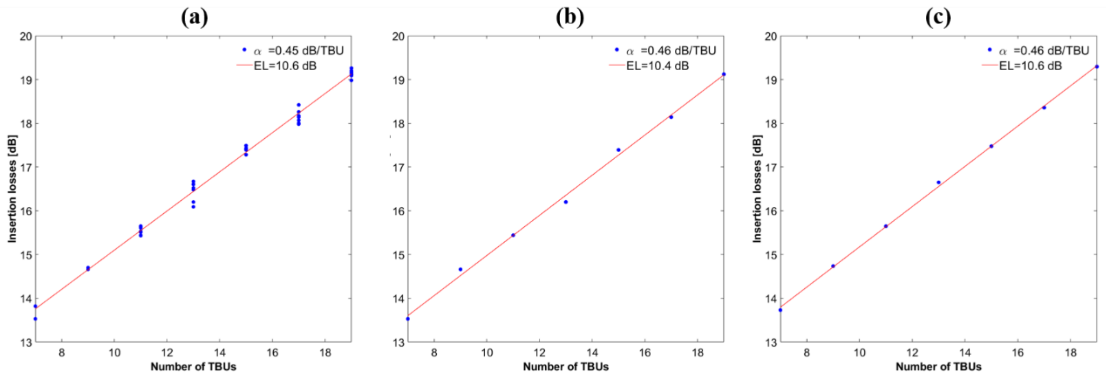

Supplementary Fig. 5: **Measured PUC insertion losses at 1550nm and fitting after cut-back measurement (odd number of connections)**: (a) for all the measured interconnects, (b) obtained from maxima spectra of each length and (c) obtained from the mean spectra of each length.

Finally, we repeated the experiment for path traversing an even number of PUCs. Similar results as for the case of odd PUC paths are shown in Figures for the even ones in Supplementary Fig. 6 and Supplementary Fig. 7. The obtained PUC losses in this case are around 0.48dB/PUC and the excess losses are around 10dB, as expected.

Overall, from the obtained results we can affirm that the PUC losses are 0.47dB ± 0.02dB, which is an enhanced result of more than 20% referring to our PUC losses of 0.6dB ± 0.1dB, reported in[6]. These include the propagation losses across the Mach Zehnder interferometer and the bend relative losses. The main contributions to this figure are the insertion losses of the MMIs present inside the PUCs.

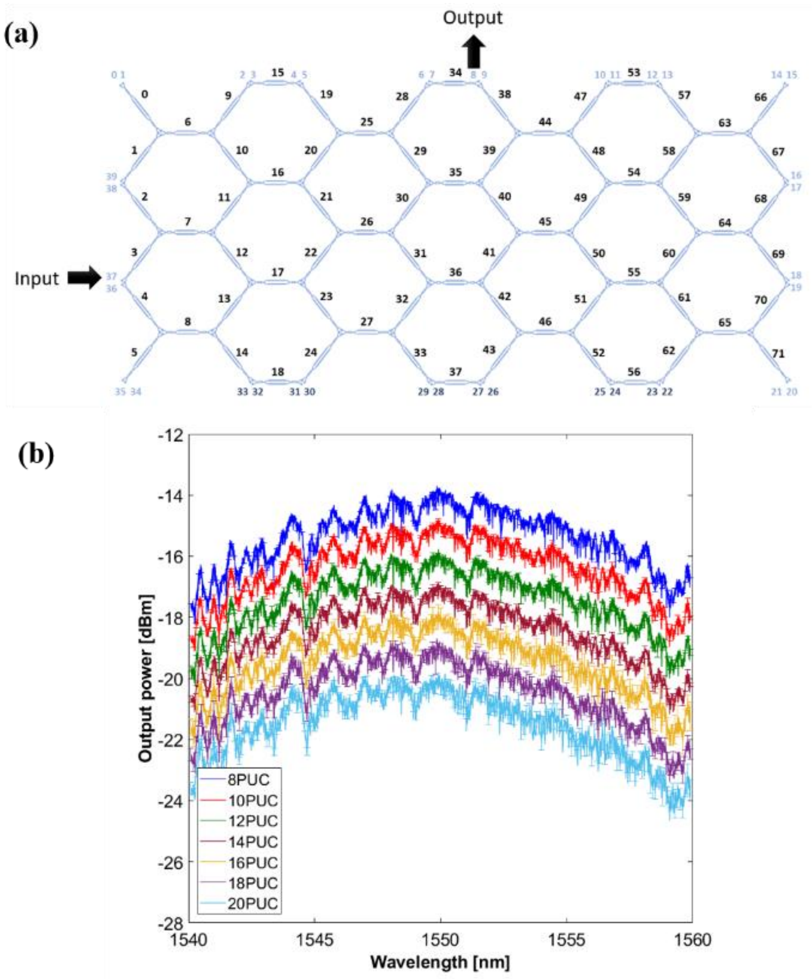

Supplementary Fig. 6: **Illustration of the cutback method for waveguide loss measurement in the waveguide mesh (even number of connections** (a) Input 37 and Output 8 ports used for the measured interconnect of different lengths and (b) Mean spectra and standard deviation.

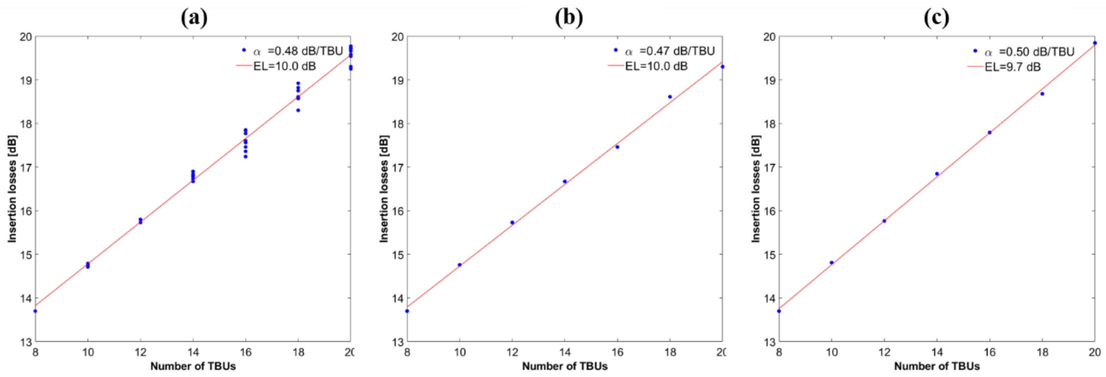

Supplementary Fig. 7: **Measured PUC insertion losses at 1550nm and fitting after cut-back measurement (even number of connections)** (a) for all the measured interconnects, (b) obtained from maxima spectra of each length and (c) obtained from the mean spectra of each length.

We also carried time domain measurements to characterize the time delay response of the waveguide mesh when programmed to implement true time delay lines of different lengths

between a pair of fixed input-output ports corresponding to the case of traversing an odd number of PUCs. Supplementary Fig. 8 shows the main measurement results:

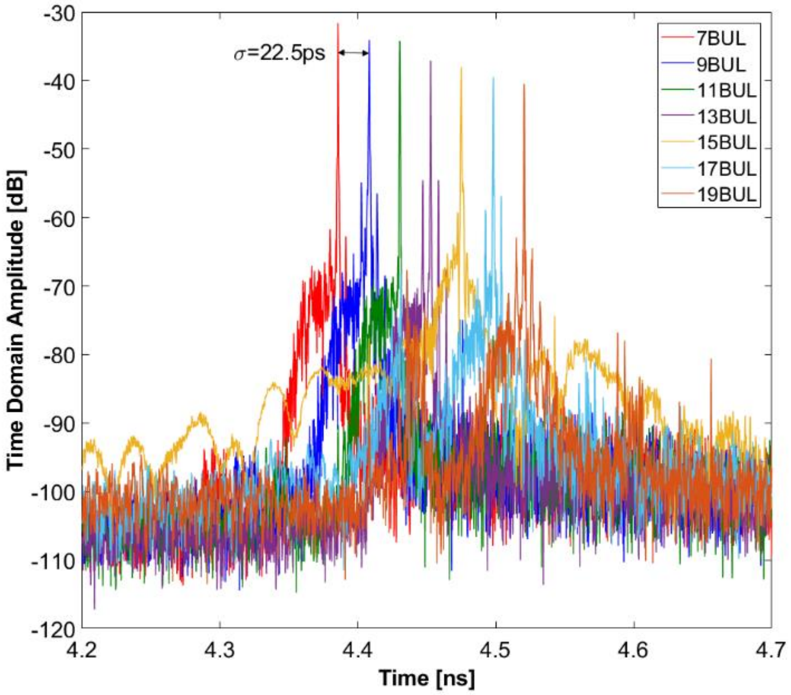

Supplementary Fig. 8: **Measured time delays for true time delay lines programmed between port 0 and port 6 and traversing an odd number of PUCs**

As it can be observed, the time delay corresponding to a step of 2BULs is 22.25 ps, which yields a final number of 11.25 ps for the propagation delay through a single PUC. Taking into consideration the refractive index of silicon, this implies a length value of 811.41µm per BUL.

Supplementary Note 4.

Implementation of tunable and reconfigurable optical filters

We programmed the general processor waveguide mesh to set different families of reconfigurable photonic filters including[7]: unbalanced Mach Zehnder Interferometers (UMZIs), Optical Ring Resonators (ORRs), Ring assisted Mach Zehnder Interferometer filters (RAMZIs), UMZI lattice filters, Coupled Resonant Waveguide filters (CROWs), Side Coupled Integrated Spaced Sequence of Optical Resonators (SCISSORS), single and double loaded RAMZI filters and simultaneous UMZI, RR and RAMZI filters.

The spectral characteristics of each configuration were measured using the same setup shown in Supplementary Figure 3. Unless otherwise specified, all the measured spectral responses in the following sections are only normalized to setup losses but are not normalized to the measured insertion losses corresponding to an integrated waveguide that connects the edge couplers to the input and output ports of one PUC in an all-pass state. These losses include the input and output edge couplers losses, the propagation losses of the mesh access paths, and the loss corresponding to one PUC, resulting in a figure of around 10.5 dB, as obtained from the interconnect measurements in Supplementary Note 2. Supplementary Table 1 provides a summary and a quick reference guide to the different configurations that we implemented and the rest of this supplementary note presents the detailed configurations and spectral responses.

**Device Nº**	Filter type	Figure
1	UMZI ΔL=2BUL	S3.1(a),<br>S3.2(a),<br>S3.3(a)
2	UMZI ΔL=4BUL	S3.1(b),<br>S3.2(b),<br>S3.3(B)
3	UMZI ΔL=6BUL	S3.1(c),<br>S3.2(c),<br>S3.3©
4	UMZI ΔL=8BUL	S3.1(d),<br>S3.2(d),<br>S3.3(d)
5	ORR L=6BUL	S3.7(a)<br>S3.7(c)<br>S3.7(e)
6	ORR L=10BUL	S3.7(b)<br>S3.7(d)<br>S3.7(f)
7	Single-Loaded RAMZI	S3.13(a)<br>S3.14(a)<br>S3.15(a)
8	Double-Loaded RAMZI	S3.13(b)<br>S3.14(b)<br>S3.15(b)
9	2nd order UMZI Lattice Filter ΔL=2BUL	S3.4(a)<br>S3.5(a)

10	3rd order UMZI Lattice Filter ΔL=2BUL	S3.4(b)<br>S3.5(b)<br>S3.6(a)
11	2nd order UMZI Lattice Filter ΔL=4BUL	S3.4(c)<br>S3.5(c)<br>S3.6(b)
12	3rd order UMZI Lattice Filter ΔL=4BUL	S3.4(d)<br>S3.5(d)
13	2nd order CROW Filter L=6BUL	S3.8(a)<br>S3.9(a)<br>S3.9(b)<br>S3.10(a)<br>S3.10(b)
14	3rd order CROW Filter L=6BUL	S3.8(b)<br>S3.9(c)<br>S3.9(d)<br>S3.10(c)<br>S3.10(d)
15	2nd order Single Channel SCISSOR Filter L=6BUL	S3.11(a)<br>S3.12(a)<br>S3.12(c)
16	3rd order Single Channel SCISSOR Filter L=6BUL	S3.11(b)<br>S3.12(b)<br>S3.12(d)
17	2nd order Single-Loaded RAMZI Lattice Filter	S3.14(c)<br>S3.15(c)
18	2nd order Double-Loaded RAMZI Lattice Filter	S3.14(d)<br>S3.15(d)
19	Simultaneous UMZI, RR and RAMZI Filters	S3.16

Supplementary Table 1: **Quick reference table for the different types of optical filters and the figures displaying their results**

UMZI Filters: We implemented UMZI filters with path unbalances given by 2, 4, 6 and 8 BULs by programming the mesh as shown in Supplementary Fig. 9 (a) to (d) respectively. The measured spectra are displayed in Supplementary Fig. 10. As it can be observed, the periodicity in the transfer function changes according to the path unbalance 43.7GHz for the 2-BUL UMZI Supplementary Fig. 10 (a)), 22.5GHz for the 4-BUL UMZI (Supplementary Fig. 10 (b)), 15GHz for the 6-BUL UMZI (Supplementary Fig. 10 (c)) and 11.2GHz for the 8-BUL UMZI (Supplementary Fig. 10 (d)). Moreover, we set coupling factors for a maximum extinction ratio of up to 30dB in each case. Total filter tuning capability along a given free spectral range (I.e. wavelength) period can be achieved by tuning a phase shifter in one of the UMZI arms. Required wavelength shifting values for a π rad cross-phase shift were Δλ=0.175 nm, Δλ=0.0907 nm, Δλ=0.0593 nm, Δλ=0.0440 nm for ΔL=2BUL, ΔL=4BUL, ΔL=6BUL and ΔL=8BUL path unbalances respectively. The measured shifting of each spectrum is shown in Supplementary Fig. 11.

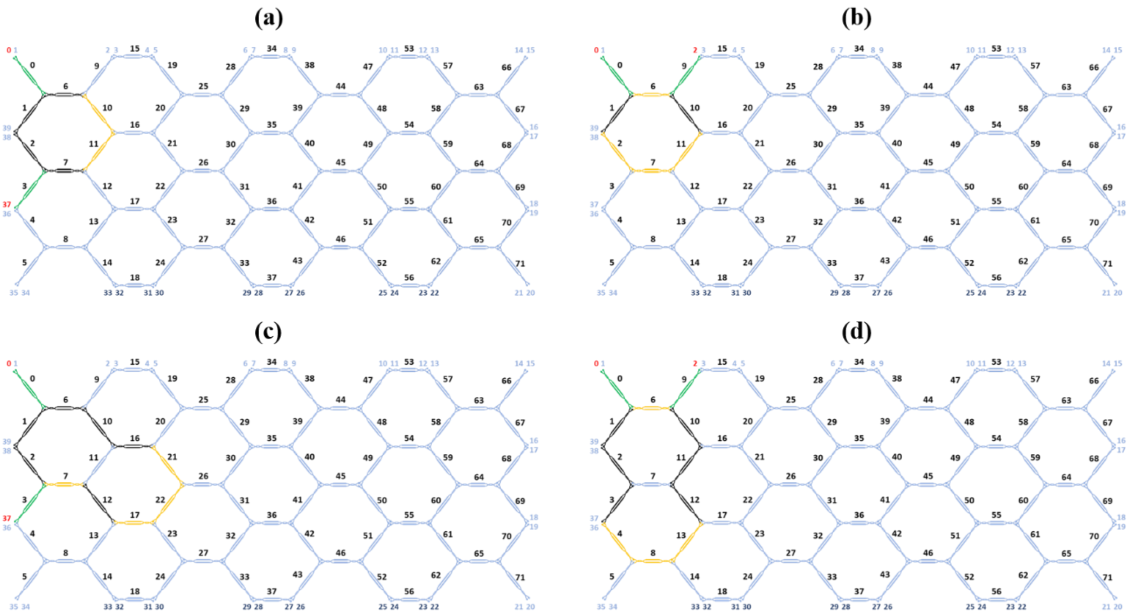

Supplementary Fig. 9. **Waveguide mesh connection diagram for the UMZIs with different path unbalances** (a) 2, (b) 4, (c), 6 and (d) 8 BULs. (Green: Tunable Coupler, Black: Cross State, Ochre: Bar state). Input and output ports are marked in red.

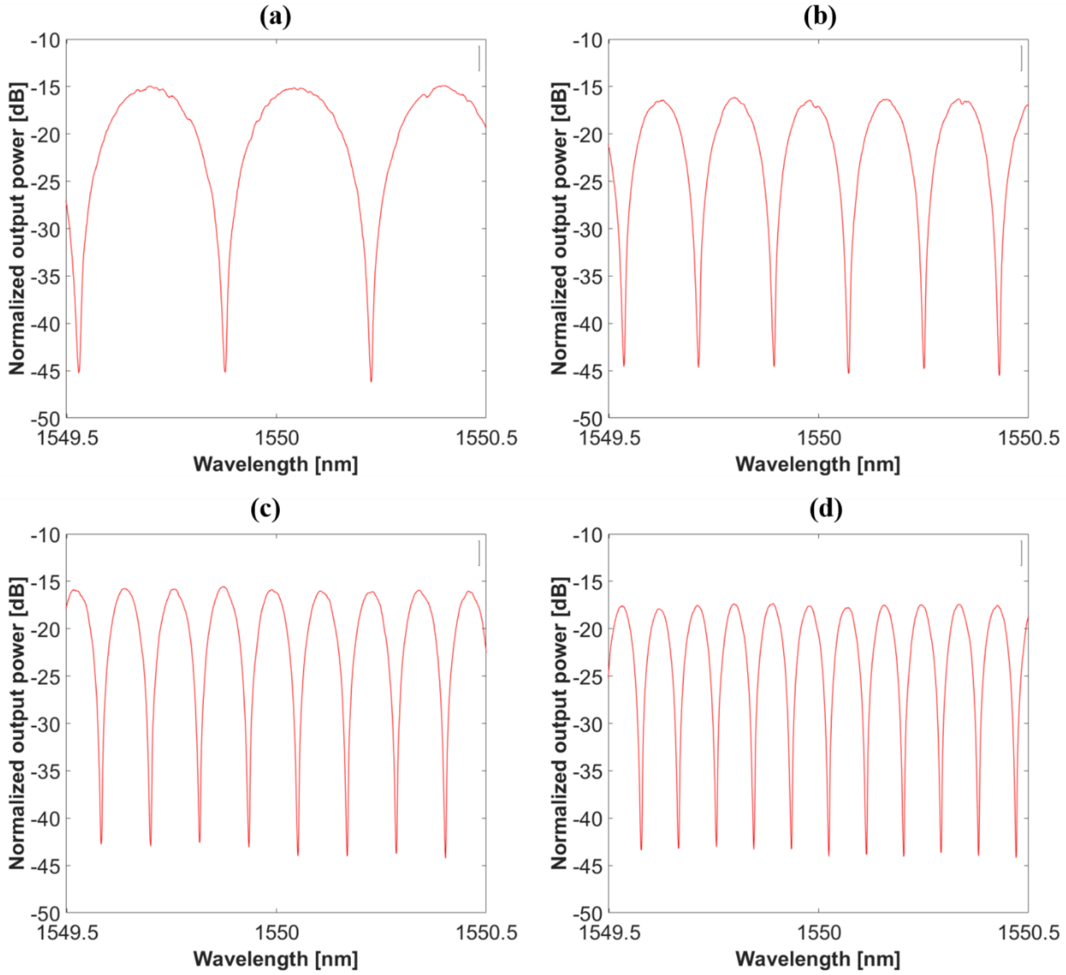

Supplementary Fig. 10. **Measured UMZI transfer functions for path unbalanced given by** (a) 2, (b) 4, (c), 6 and (d) 8 BULs.

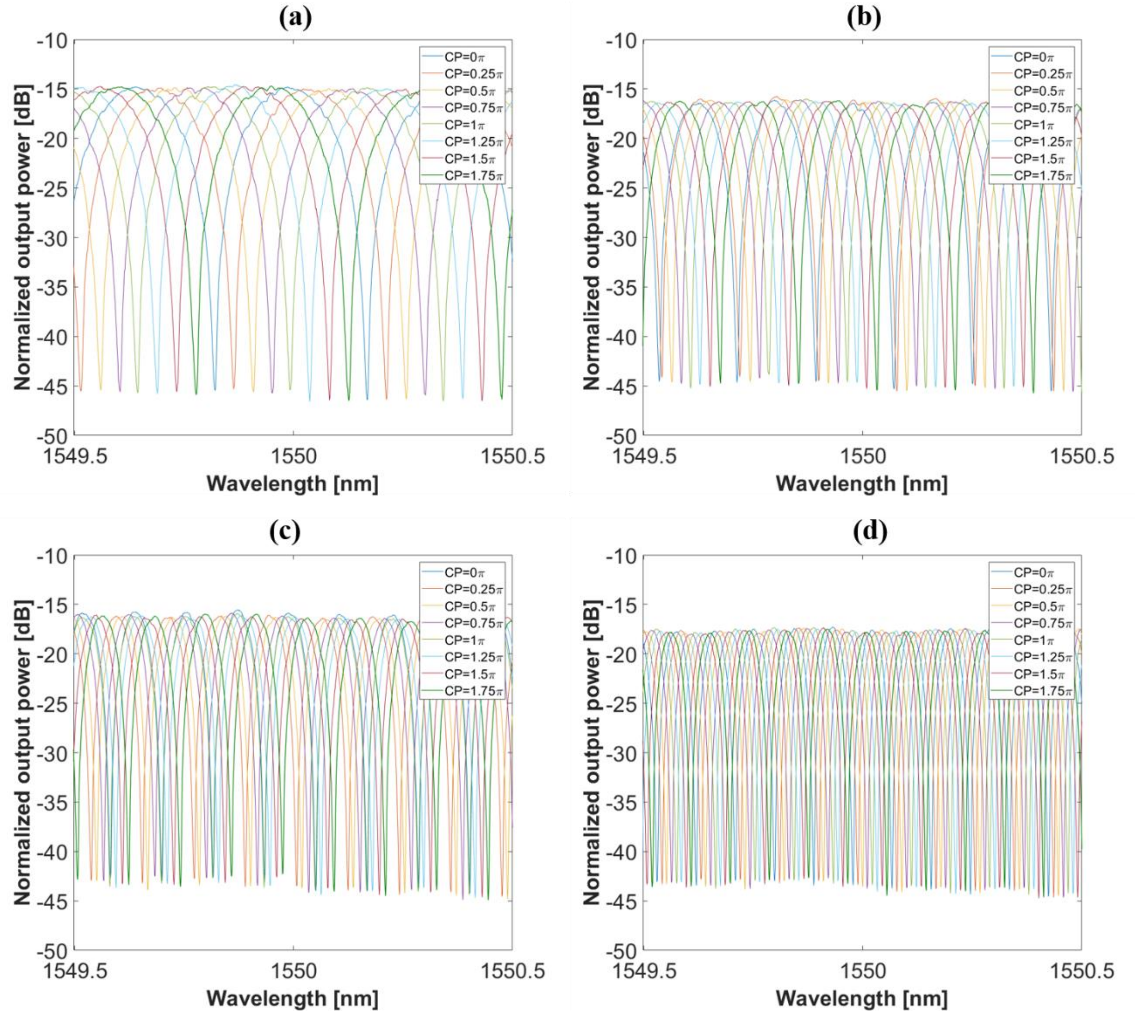

Supplementary Fig. 11: **Measured tunability for the UMZIs with path unbalances given by** (a) 2, (b) 4, (c) 6 and (d) 8 BULs for fixed coupling factor and different cross phases.

UMZI Lattice Filters: We also programmed and implemented more complex lattice filters by serially cascading UMZI units. We have only measured filters up to the 4th order, although, due to the mesh size, up to the 7th order could be synthesized. Supplementary Fig. 12 (a)-(d) shows the mesh programming for 2nd and 3rd order lattice filters, with ΔL=2BUL, and for 2nd and 3rd order lattice filters, with ΔL=4BUL respectively.

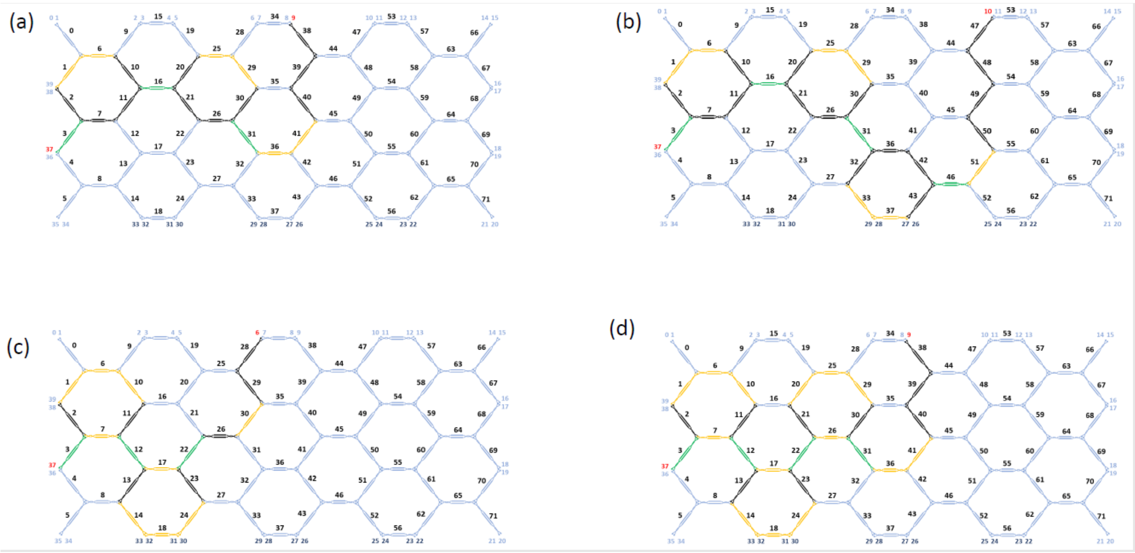

Supplementary Fig. 12: **Waveguide mesh connection diagram for the 2nd and 3rd order UMZI Lattice filters with path unbalances given by 2 BUL and 4BUL** (a) Second order UMZI lattice filter and path unbalance 2 BUL. (b)) Third order UMZI lattice filter and path unbalance 2 BUL, (c)) Second order UMZI lattice filter and path unbalance 4 BUL. (d)) Third order UMZI lattice filter and path unbalance 3 BUL. (Green: Tunable Coupler, Black: Cross State, Ochre: Bar state). Input and output ports are marked in red.

Supplementary Fig. 13 (a) and (b) show the spectra of 2nd and 3rd-order lattice filters, respectively, with ΔL=2BUL cascaded UMZIs and different values of the tunable couplers coupling constants, s. Supplementary Fig. 13 shows the spectra of 2nd, 3rd, and 4th order lattice filters respectively with ΔL=4BUL cascaded UMZIs and different values of the tunable coupler coupling constants. We measured the tunability for fixed coupling factors and different phase shifter tunings. Supplementary Fig. 14 shows the tunability of two of them.

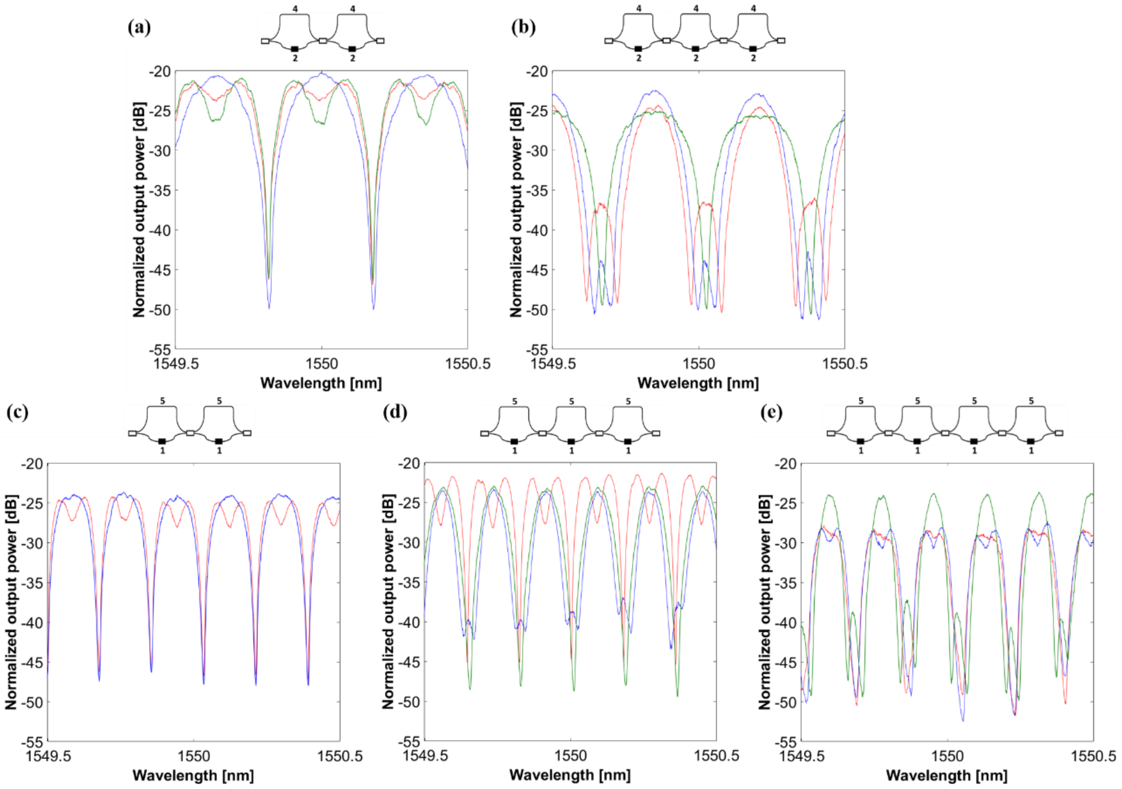

Supplementary Fig. 13: **Schematic and measured transfer functions for lattice filters composed by cascaded UMZIs for different values of coupling constants** (a) 2nd order, ΔL=2BUL, (b) 3rd order, ΔL=2BUL, (c) 2nd order, ΔL=4BUL, (d) 3rd order, ΔL=4BUL and (e) 4th order, ΔL=4BUL. (white square: tunable coupler, black square: phase shifter).

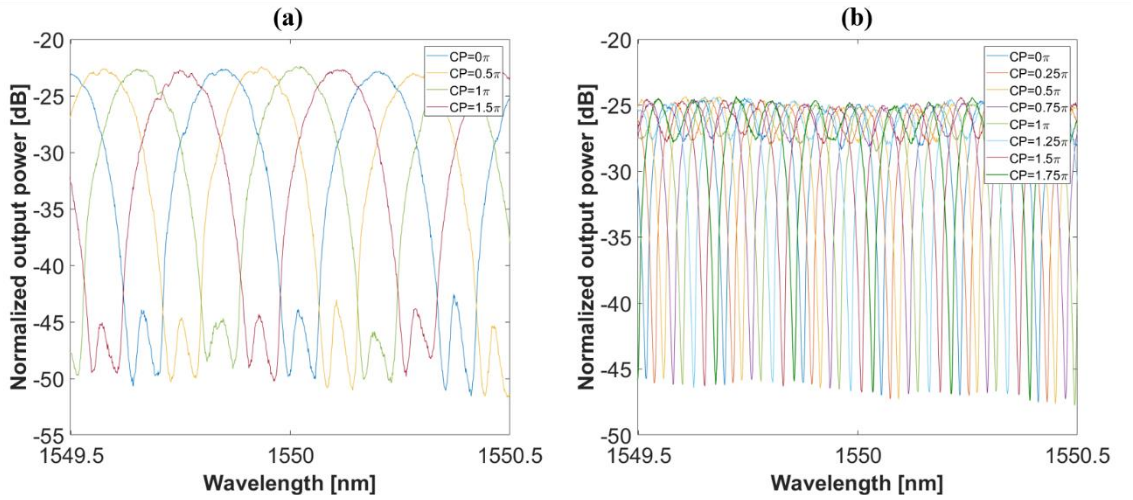

Supplementary Fig. 14: **Measured tunability for lattice filters composed by cascaded UMZIs** (a) 3rd order, ΔL=2BUL and (b) 2nd order, ΔL=4BUL, for fixed coupling factor and different cross phases

Optical Ring Resonators: It is possible to synthesize optical ring resonators (ORRs) with many different values of cavity lengths. As a proof of concept, we programmed single cavity optical ring resonators with cavity lengths given by 6 (Supplementary Fig. 15 (a)) and 10 BULs (Supplementary Fig. 15 (b). As expected, the periodicity in the transfer function changes according to ORR cavity length: 15GHz for the 6-BUL cavity length in Supplementary Fig. 15 (c)**¡Error! No se encuentra el origen de la referencia.¡Error! No se encuentra el origen de la referencia.** and 8.75GHz for the 10-BUL cavity length in Supplementary Fig. 15 (d)**¡Error! No se encuentra el origen de la referencia.**. Moreover, we chose coupling factors for a maximum extinction ratio of around 25dB in the reflection case and around 10dB in the transmission case. Finally, we also demonstrate the tunability capability of these filters by fixing the coupling factors and changing the cross-phases Supplementary Fig. 15 (e) for 6-BUL and Supplementary Fig. 15 (f) for 10-BUL cavity lengths). Wavelength shifts of 0.0582nm and 0.0335nm per π-rad cross phase were measured for 6-BUL and 10-BUL cavity lengths respectively.

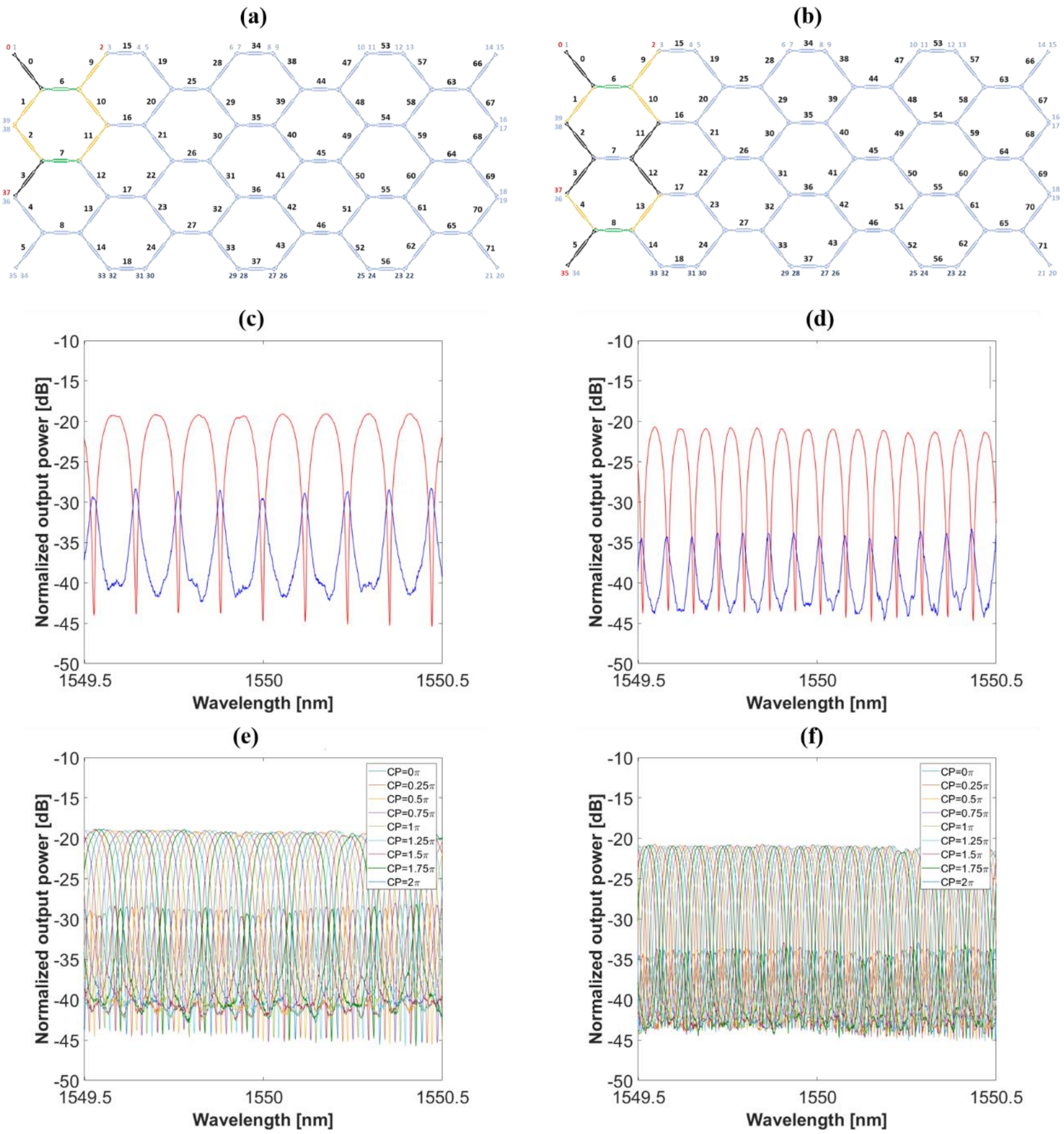

Supplementary Fig. 15: **Waveguide mesh connection diagram for the ORRs** (a) 6-BUL and (b) 10-BUL cavity lengths (Green: Tunable Coupler, Black: Cross State, Ochre: Bar state). Input and output ports are marked in red. Transmission and reflection measured transfer functions for the ORRs with cavity lengths given by (c) 6-BUL and (d) 10-BULs. Tuning capability measured for fixed coupling factor and different cross phases for cavity lengths given by (e) 6-BUL and (f) 10-BULs.

CROW Filters: Supplementary Fig. 16 (a) and (b) show the mesh programming for the implementation of a second and a third-order 6BUL CROW filter respectively. The results for their spectral responses are displayed in Supplementary Fig. 17 (2nd order in (a) and (b) and 3rd order in (c) and (d)) in both reflection (left column) and transmission (right column) configurations, for different coupling factors, and therefore, showing different transfer functions.

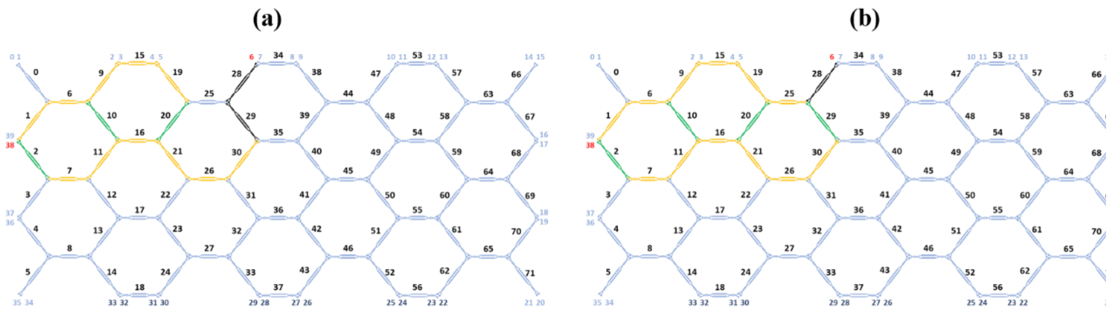

Supplementary Fig. 16: **Waveguide mesh connection diagram for the 2nd and 3rd order 6BUL CROW Filters** (a) second order filter (b) third order filter. (Green: Tunable Coupler, Black: Cross State, Ochre: Bar state). Input and output ports are marked in red.

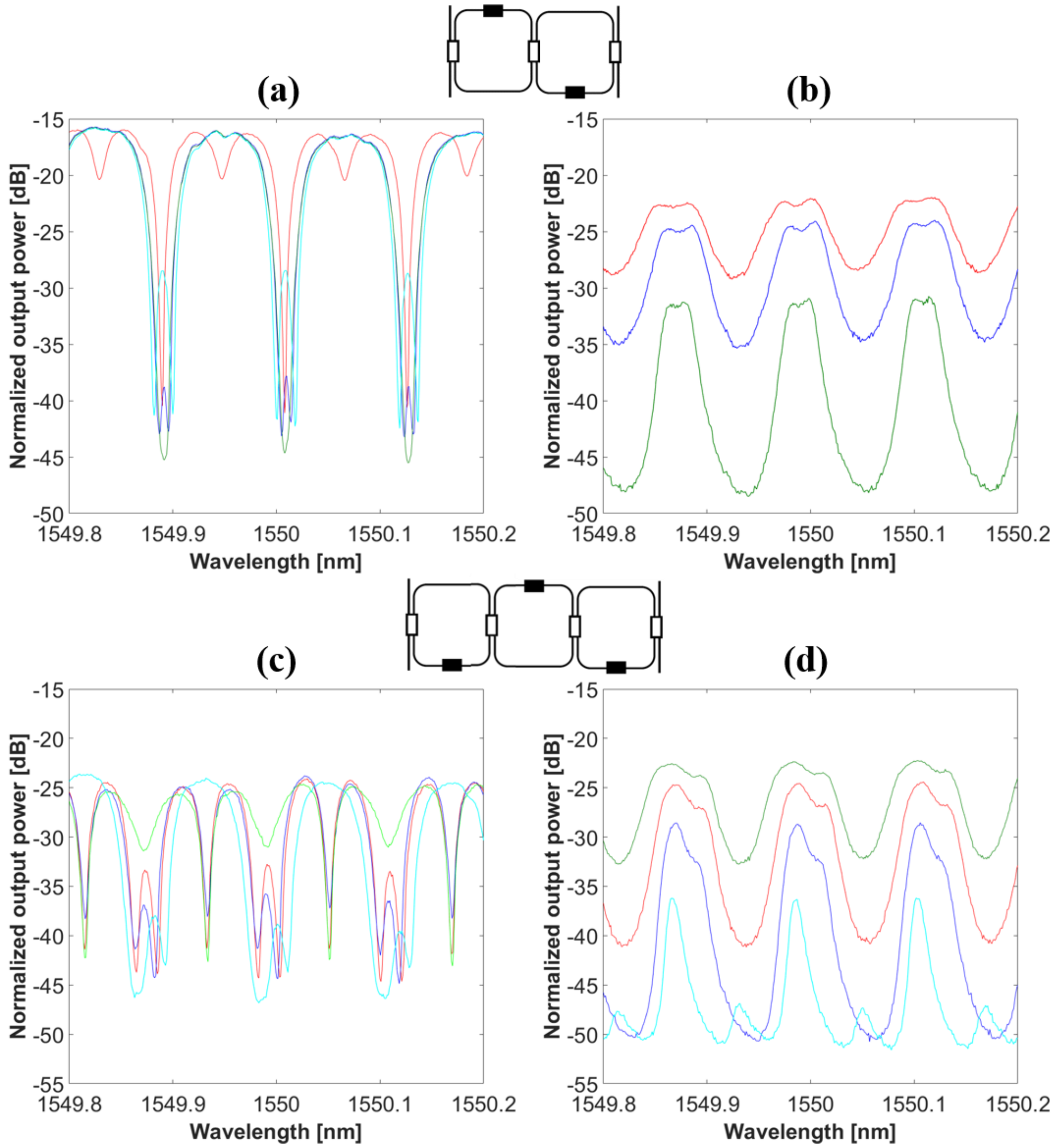

Supplementary Fig. 17: Schematic and measured transfer functions of CROW structures composed by cascaded 6-BUL ORRs for different values of coupling constants (a) reflection and (b) transmission 2nd

order, (c) reflection and (d) transmission 3rd order (white square: tunable coupler, black square: phase shifter).

As in the previously reported filters we also measured and demonstrated their tunability capability by fixing the coupling factors and changing the cross-phases for all the measured CROW filters. Some examples are displayed in Supplementary Fig. 18.

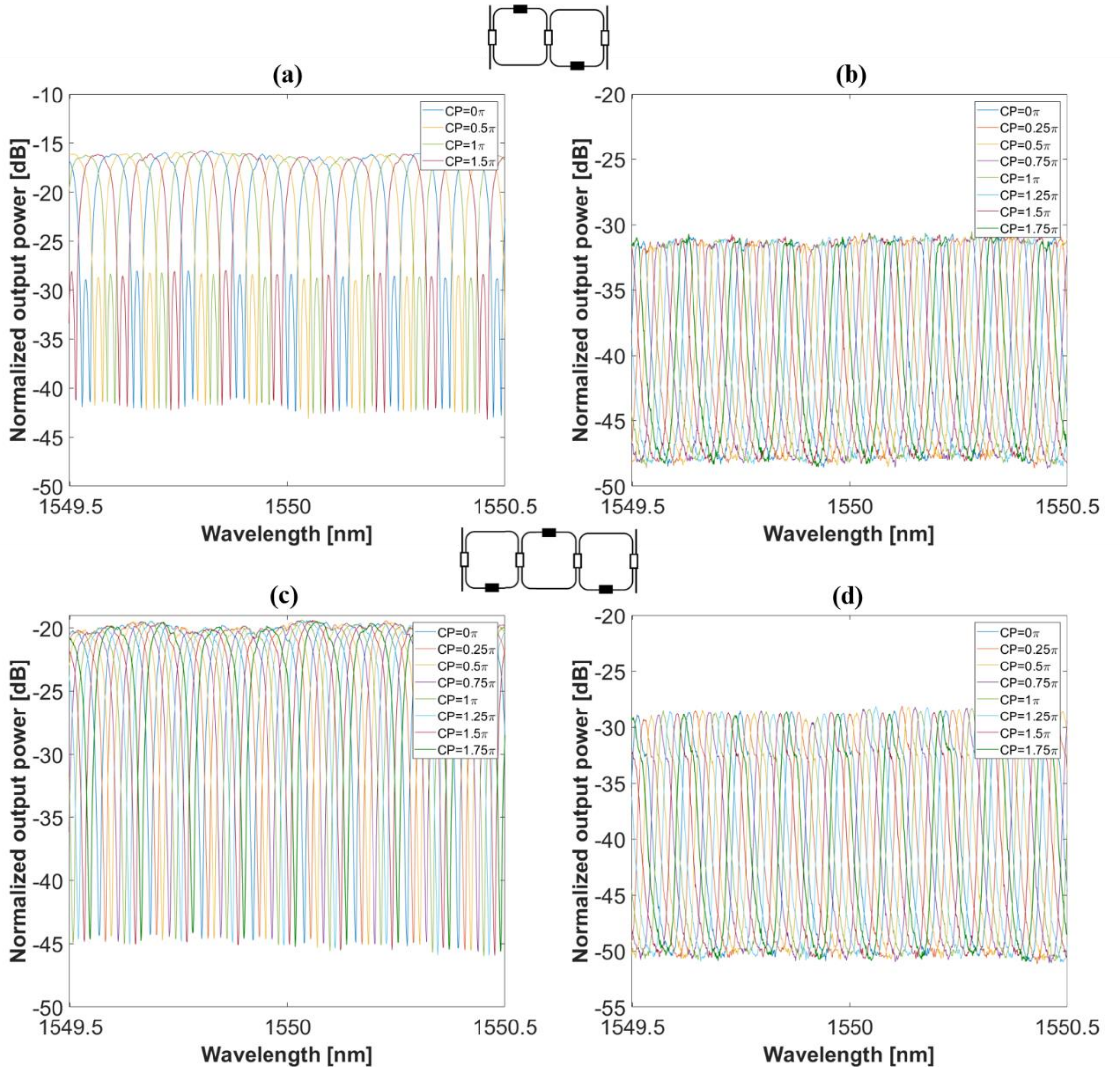

Supplementary Fig. 18**: Schematic and measured tuning for fixed coupling factor and different cross phases of CROW structures composed by cascaded 6-BUL ORRs for a fixed value of coupling constants** (a) reflection and (b) transmission 2nd order, (c) reflection and (d) transmission 3rd order (white square: tunable coupler, black square: phase shifter).

SCISSOR Filters: We implemented both 2nd and 3rd-order 6BUL SCISSOR structures by suitable programming of the photonic core as shown in Supplementary Fig. 19.

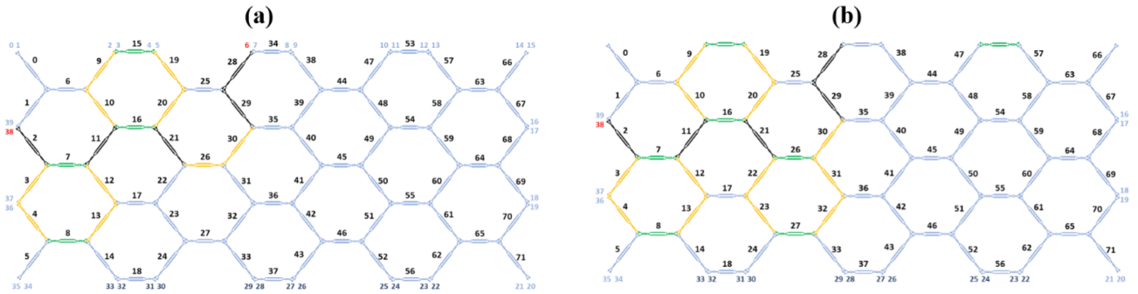

Supplementary Fig. 19: **Waveguide mesh connection diagram for the 2nd and 3rd order 6BUL SCISSOR Filter**s. (a) second order filter, (b) third order filter (Green: Tunable Coupler, Black: Cross State, Ochre: Bar state). Input and output ports are marked in red.

The results for their spectral responses and the resonance tuning by acting over intracavity phase shifters are displayed in Supplementary Fig. 20 (2nd order in (a) and (c) and 3rd order in (b) and (d)), for different coupling factors showing different transfer function shapes.

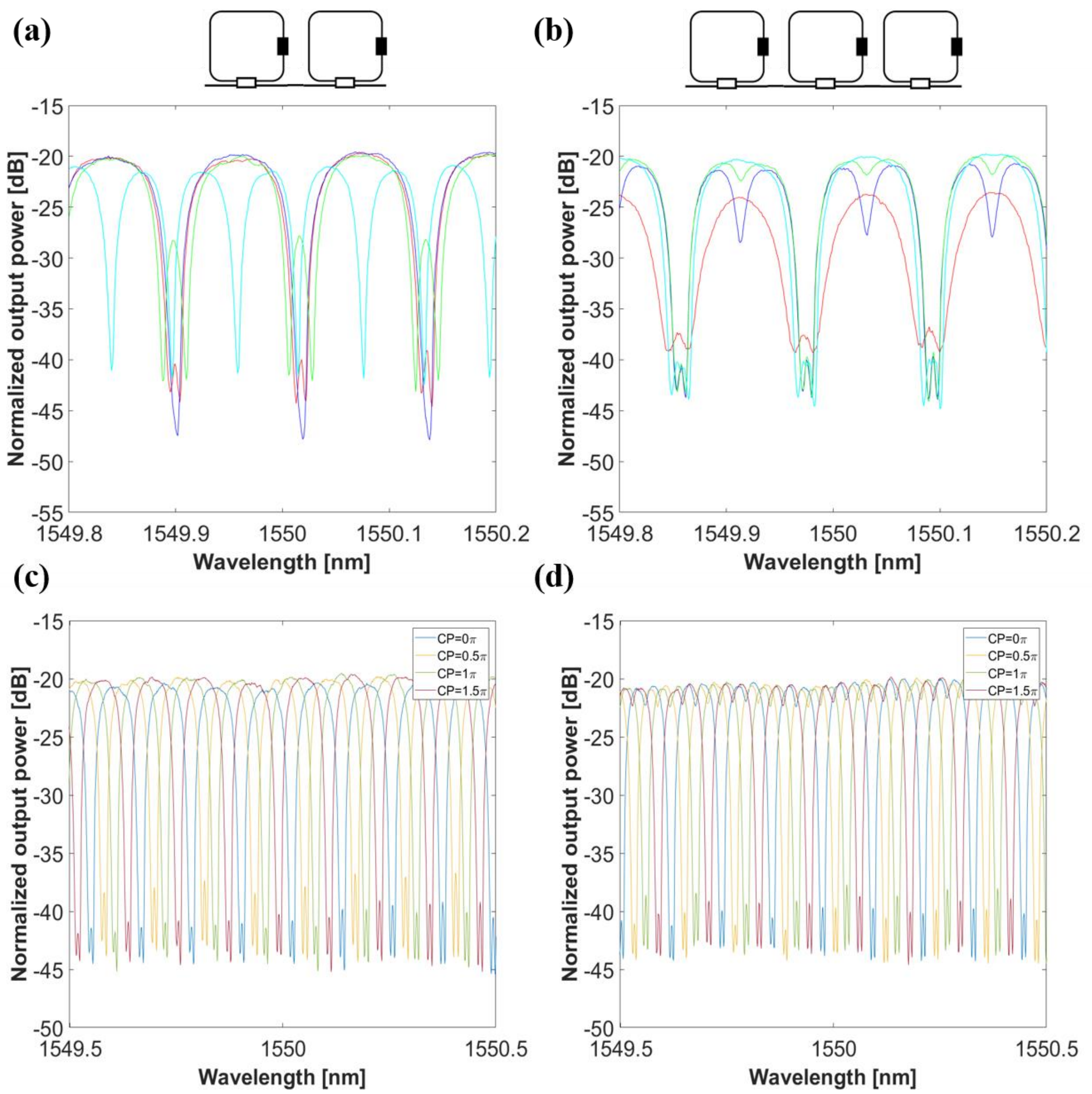

Supplementary Fig. 20: ***Schematic and measured transfer functions of SCISSOR structures composed by cascaded 6-BUL ORRs for different values of coupling constants*** (a) 2nd order and (b) 3rd order, and resonance tuning by means of different cross phases for a fixed coupling factor (c) 2nd order and (d) 3rd order (white square: tunable coupler, black square: phase shifter).

RAMZI Filters: The last type of complex filters we measured and demonstrated by synthesizing the mesh, are ring-loaded UMZIs (RAMZIs). In this case, we programmed simple single (Supplementary Fig. 21 (a)) and double-loaded (DL) (Supplementary Fig. 21 (b)) RAMZIs. Also, 2nd order single and double-loaded RAMZI filters were programmed and implemented.

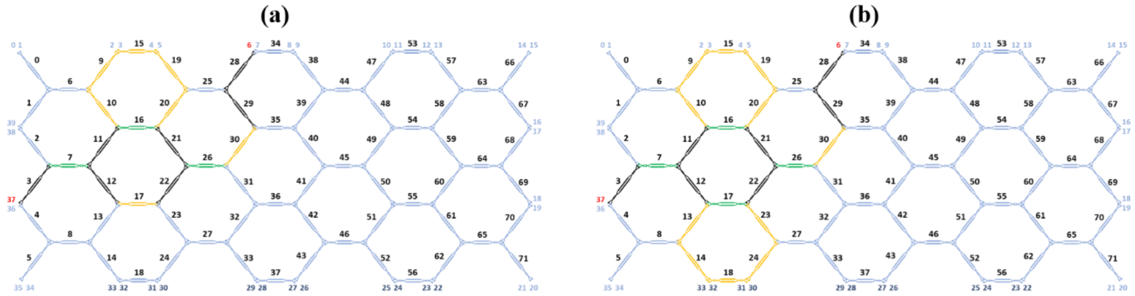

Supplementary Fig. 21. **Waveguide mesh connection diagram for single and double-loaded RAMZI Filters.** (a) single loaded RAMZI. (b) double loaded RAMZI *(Green: Tunable Coupler, Black: Cross State, Oc hre: Bar state). Input and output ports are marked in red.*

Supplementary Fig. 22 shows the transfer functions for each of these structures and different values of the ring coupling constants.

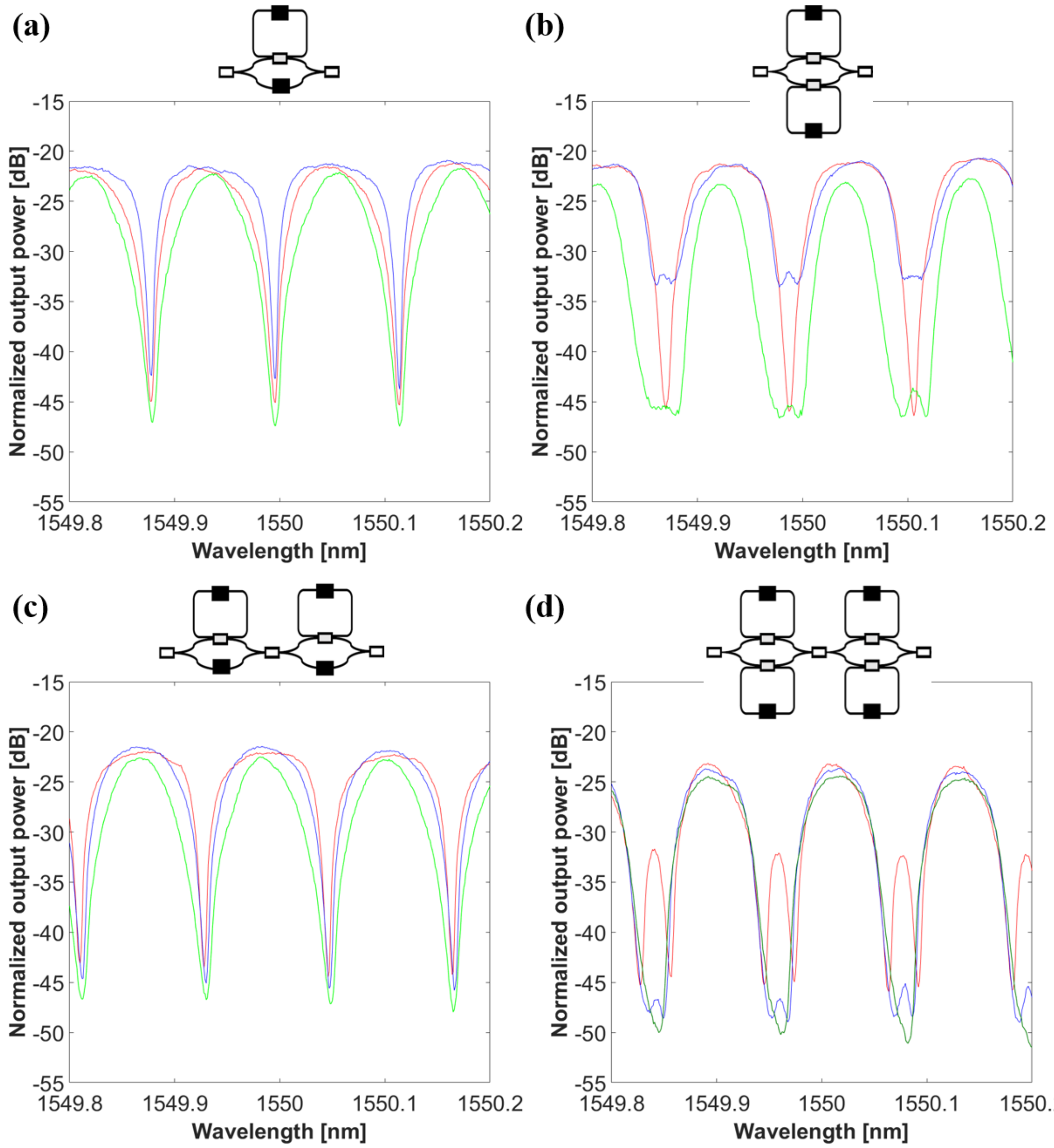

Supplementary Fig. 22: **Schematic and measured transfer functions of RAMZIs structures composed by cascaded UMZIs loaded with ORRs, for different values of coupling constants** (a) single loaded and (b) double loaded, (c) 2nd order single loaded and (d) 2nd order double loaded (white square: tunable coupler, black square: phase shifter).

Again, tunability capability was demonstrated by measuring the spectral response shifting by tuning the cross phases (CPs) for fixed coupling constants. Supplementary Fig. 23 shows some examples of measured filters.

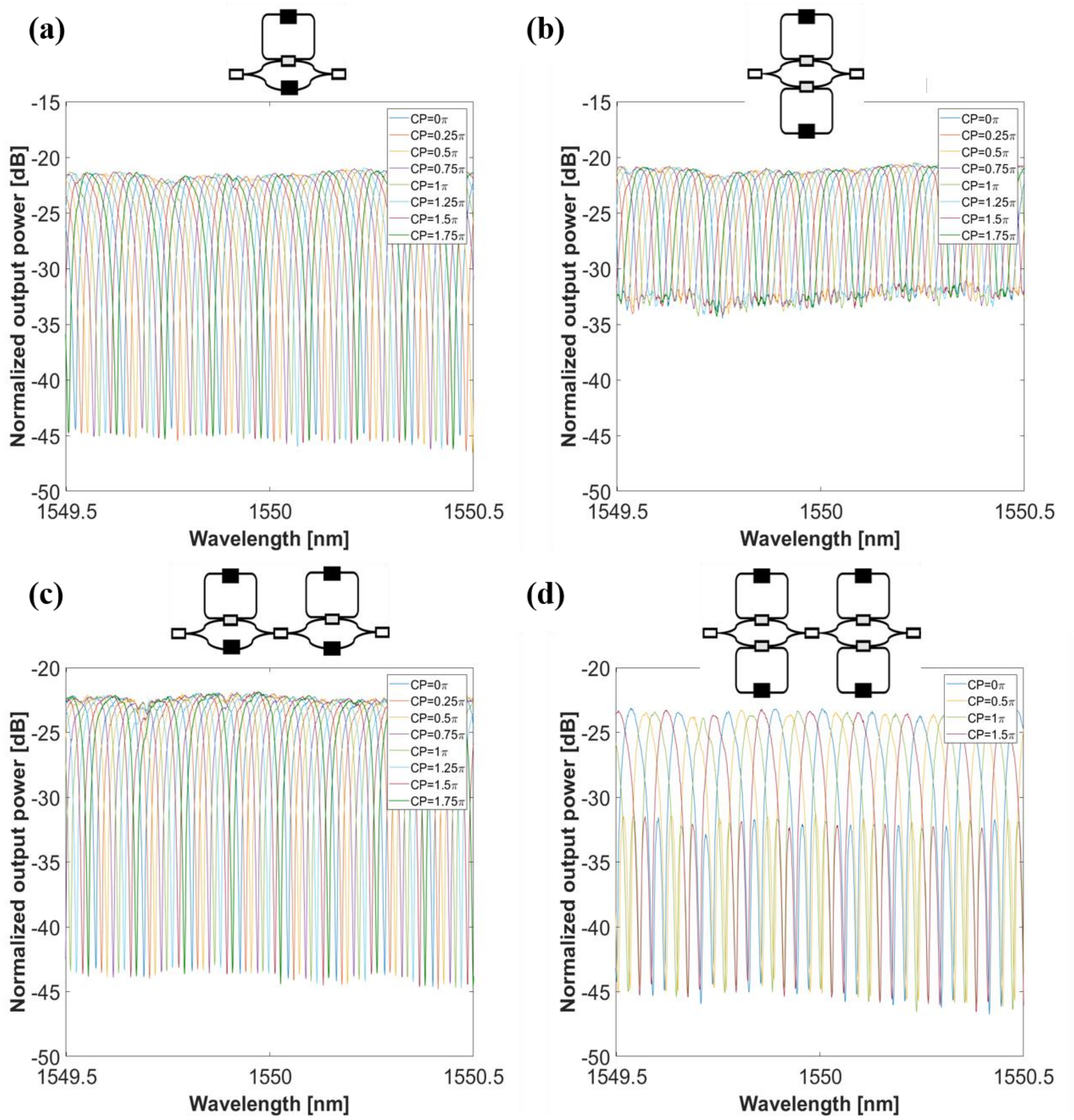

Supplementary Fig. 23. **Schematic and measured tuning for fixed coupling factor and different cross phases of RAMZIs structures composed by cascaded UMZIs with loaded 6-BUL ORRs for a fixed value of coupling constants** (a) single loaded and (b) double loaded, (c) 2nd order single loaded and (d) 2nd order double-loaded (white square: tunable coupler, black square: phase shifter).

Simultaneous UMZI, DL-RAMZI, and ORR Filters: Finally, with the aim of demonstrating the simultaneity capability allowed by the processor core, we synthesized three filters in the mesh at the same time, and measured the optical crosstalk. As it can be observed in Supplementary Fig. 24, a UMZI with 2 BUL arm imbalance, a double-loaded single RAMZI (DL-RAMZI, 6BUL cavities) and an ORR (6 BUL cavity) were set in the mesh. Then, with the input light at the UMZI input port (Port 0), we measured the transmission response in each output of these three filters. After, we made the same test with the input light at the DL-RAMZI input (Port 35) and finally, with the input light at the ORR input (Port 14). Results of the optical crosstalk are shown in Supplementary Fig. 24 (b), (c) and (d) respectively. As it can be noticed from the figures, up to 60dB of optical rejection was obtained.

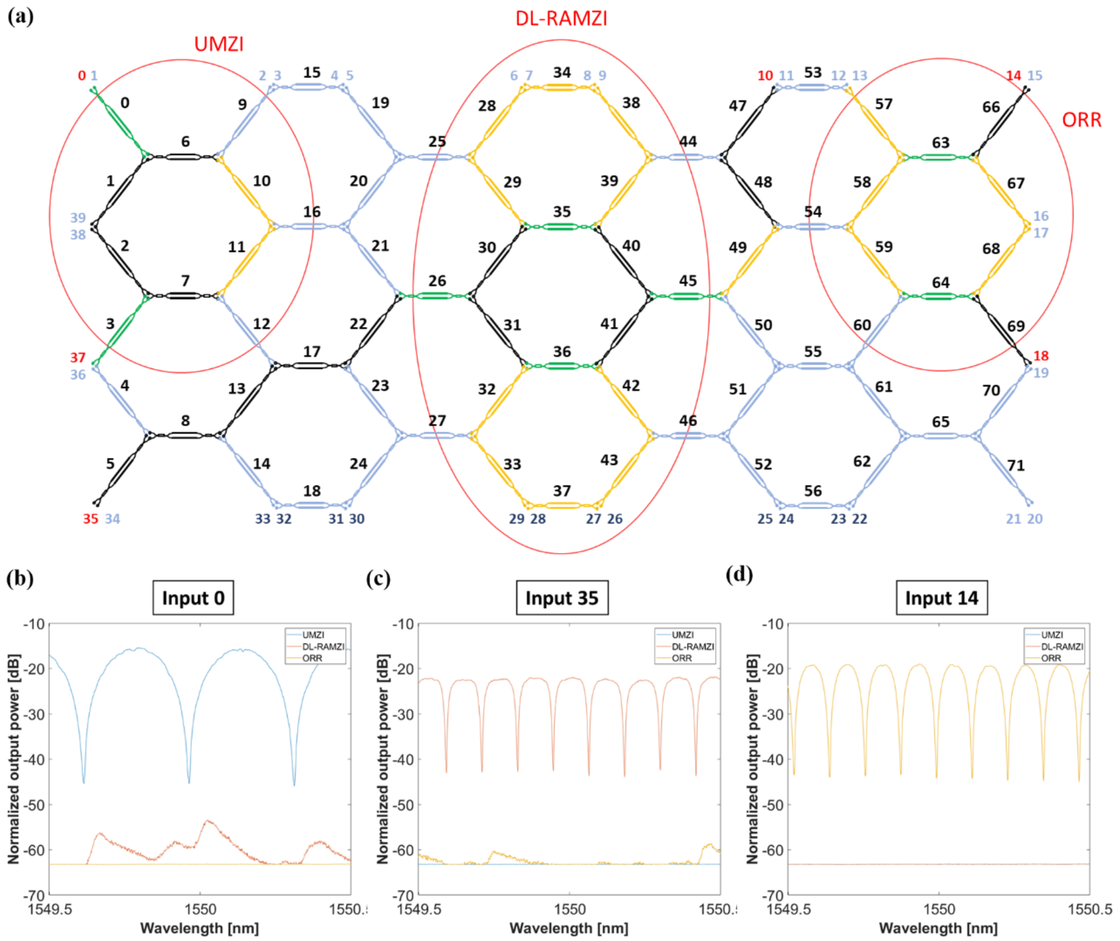

Supplementary Fig. 24 **Example of multiple filter implementation in the same waveguide mesh.** (a): Waveguide mesh connection diagram for the synthesized 2 BUL UMZI, double-loaded (6 BUL) RAMZI, and ORR (6 BUL) filters at the same time. Input and output ports are marked in red, (b) Spectral response and crosstalk level for the UMZI filter, (c) Spectral response and crosstalk level for the RD-RAMZI filter, (d) Spectral response and crosstalk level for the ORR filter

Supplementary Note 5.

Implementation of tunable and reconfigurable RF filters and phase shifters

RF-photonic signal filtering is probably the most widespread application in integrated MWP processors. Specifically, the combination of flexible reconfigurable optical cores and modulation and photodetection subsystems, enable the design of fully integrated tunable RF-photonic devices by down-conversion of the spectral characteristics of the photonic filter to the RF band[8-10]. It is important to highlight here that, when compared to application-specific photonic circuits, the programmable processor suffers from extra excess losses due to the waveguide lattice mesh, reducing the total RF gain of the filter. To overcome this limitation, the integration of optical amplifiers in the system can be considered[11].

To implement a microwave photonic filter, different modulation and detection schemes can be employed to increase the tunability range, the dynamic range, the gain, and the noise figure of the whole system[10]. The optical filters we employ for down conversion into the microwave photonic filtering are the 3rd order UMZI (ΔL=2BUL) lattice filter and the 2nd order double-loaded RAMZI filter demonstrated in Supplementary Note 3. Down conversion and subsequent RF filter is obtained from the beating of one of the RF sidebands, and the optical carrier.

Since we required single-sideband (SSB) modulation we employed in our experimental setup a dual-drive modulator (in single-drive configuration) in combination with a wave-shaper implementing a stop-band filter to mitigate one of the RF sidebands, avoiding the use of an electrical 90° hybrid. Moreover, we used an MWP system operating in the so-called self-beating mode that works in a self-homodyne fashion by sharing the same laser source for information bearing and local oscillator tasks[12].

The final scheme used for mapping the optical filter responses to the RF domain is based on carrier suppression, single-sideband, and self-beating techniques. Therefore, to demonstrate the photonic microwave filtering capability of the mesh, we followed the setup scheme shown Supplementary Fig. 25.

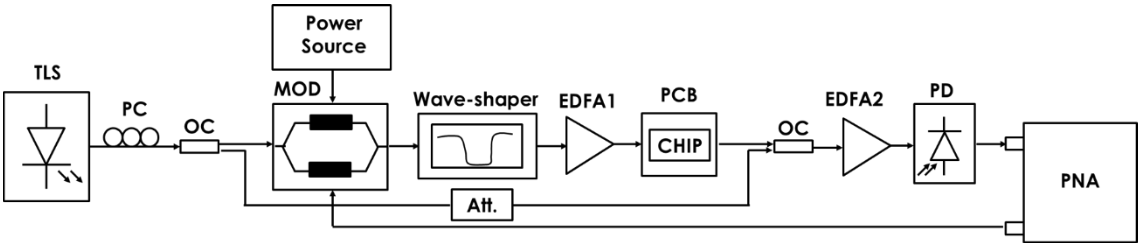

Supplementary Fig. 25**: Passive setup used to measure the spectra of the different optical interconnects**.

First, an optical carrier emitted by a tunable laser source (TLS), and a vector network analyzer (PNA, Optical Network Analyzer N4373C from Agilent) is used to modulate the electro-optic modulator biased at the quadrature bias point (QB). A dual-drive modulator (MOD) Sumitomo T.DEH1.5-40-ADC was used as a modulator, in combination with a wave-shaper 4000s from Finisar, implementing a stop-band filter to implement the carrier suppression and single-sideband modulation. In addition, we used two optical couplers (OC) at the input (50/50) and output (50/50) to implement the self-beating technique. The SSB modulated signal is amplified by an AEDFA-23-B-FA erbium-doped fiber amplifier (EDFA) from Amonics and introduced to the optical integrated filter synthesized in the photonic mesh. Once the signal is outside the chip, it

is amplified again with another AEDFA-23-B-FA EDFA for compensating the losses suffered in the integrated chip, and photo detected (XPDV2320R-VF-VA from Finisar) and sent to the microwave network analyzer (PNA) to measure the transmission response.

Supplementary Fig. 26 shows optical responses (left column) and the resulting down-converted RF filter shapes (right column) measured up to 50GHz and mapped with the 3rd order UMZI (ΔL=2BUL) lattice filter (Supplementary Fig. 26 (a), (b)) and the 2nd order double-loaded RAMZI filter (Supplementary Fig. 26 (c), (d)). As it can be observed, microwave photonic notch filters with high peak rejection in RF power (40 dB and 30dB respectively) are obtained and free spectral ranges of 44GHz and 15GHz, respectively, are demonstrated. The RF filter responses are normalized by the non-flat response of the system (modulator + EDFA + photodetector, without the optical filter).

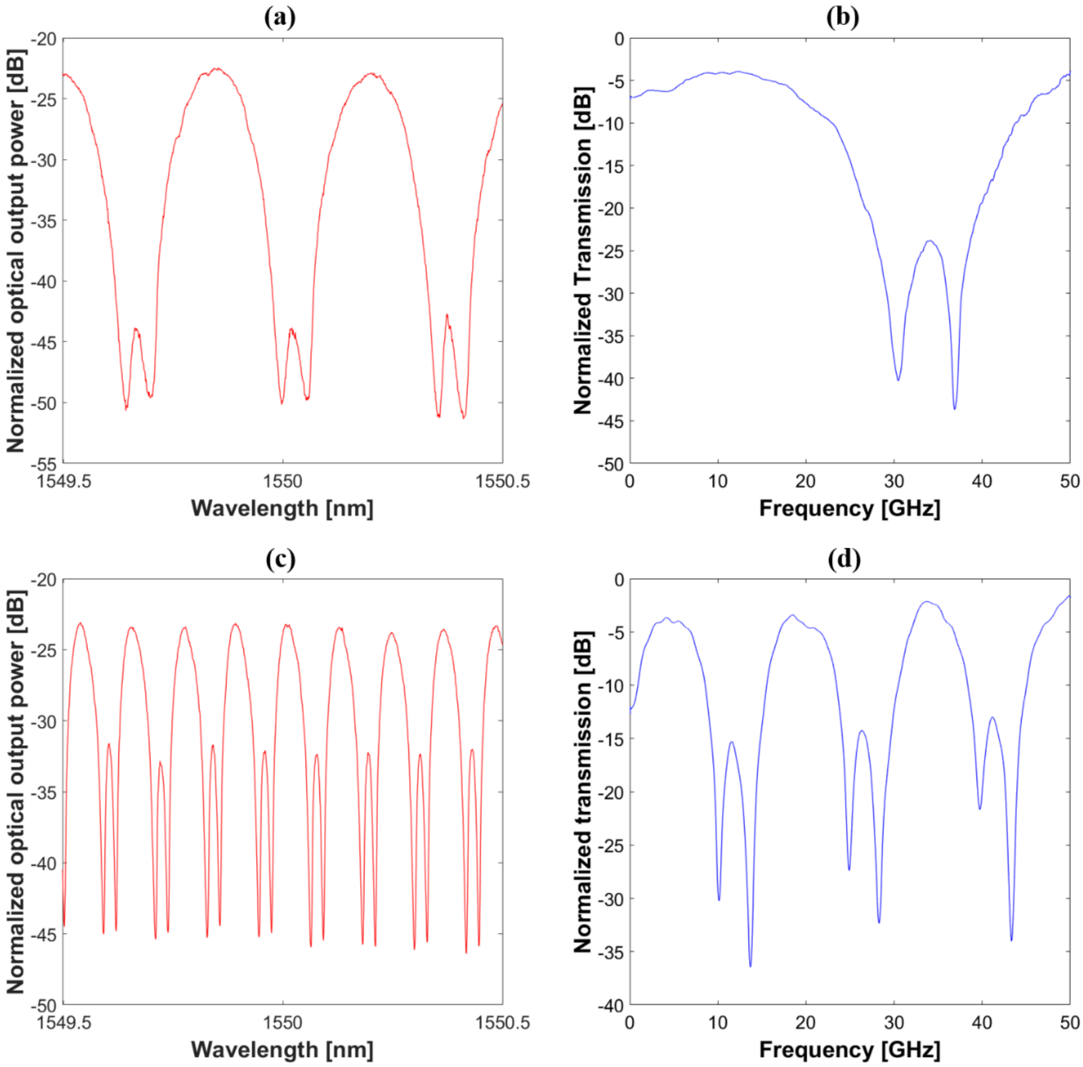

Supplementary Fig. 26: **Optical (left column) and microwave photonic filter responses (right column) of 3rd order UMZI (ΔL=2BUL) lattice and 2nd order double-loaded RAMZI filters** (a) 3rd order UMZI (ΔL=2BUL) lattice optical filter response, (b) corresponding RF down converted microwave photonic response, (c) 2nd order double-loaded RAMZI optical filter response. (d) corresponding RF down converted microwave photonic response. Microwave responses are normalized to the modulator, the EDFAs, and the photodetector link response.

For tunability, by sweeping the relative position of the carrier concerning the spectral trace of the optical filter, we can induce the same tuning to the beating of the filtered RF sideband signal.

Although this can be done, by tuning the spectral response of the filter, in this case, one of the mechanisms used to tune the MWP filter is the carrier wavelength sweeping (wavelength tuning). The other mechanism is carried out by making use of the photonic mesh itself to implement an equivalent RF phase shifter. The optical filter is tuned by sweeping the cross-phase shifters, as demonstrated in Supplementary Note 3, and therefore, the optical phase tuning is translated to an electrical phase tuning of the microwave photonic filter after the photodetection. Supplementary Fig. 27 shows the tunability results obtained for both MWP filters measured. As can be observed, sweeping the cross-phase shifters leads to a higher tunability range with respect to the wavelength shifting technique.

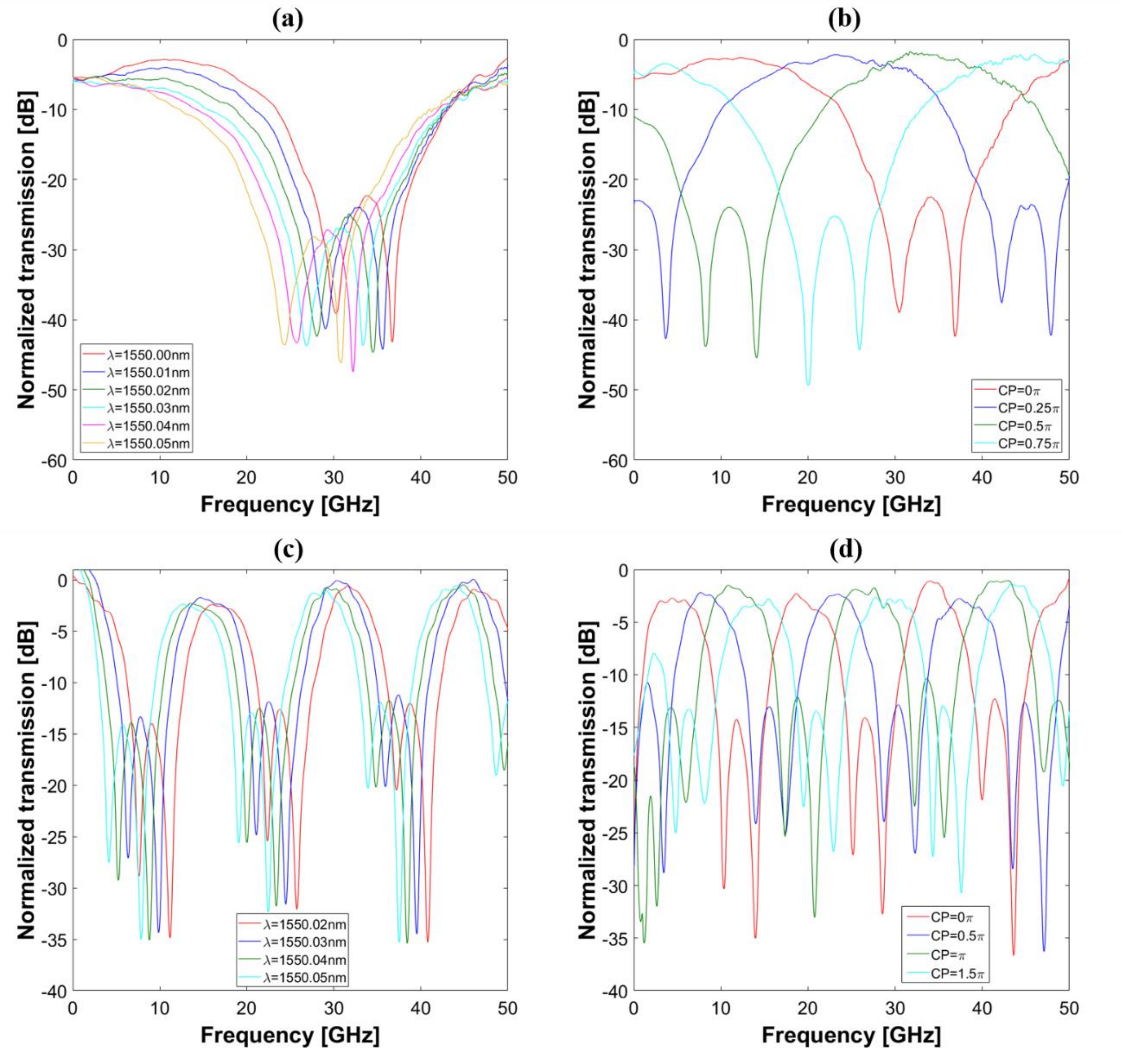

Supplementary Fig. 27: **MWP filter tunability feature by carrier wavelength shifting (left column) and cross-phase sweeping (right column) demonstrated in the 3rd order UMZI (ΔL=2BUL) lattice** filter (a)-(b) and 2nd order double-loaded RAMZI filter (c)(d). Responses are normalized to the modulator, the EDFAs, and the photodetector link response.

Supplementary Note 6.

Optical generation of MM-wave CW signals

RF Many emerging broadband low-latency applications in wireless communication and sensor networks operate in the high portion of the millimetre (mm) wave band (i.e., above 30 GHz)[13]. For example, the 5G New Radio (NR) standard has selected in its frequency range two (FR2) the use of the frequencies in the three next ranges: 24.25 - 29.50 GHz, 37 - 43.5 GHz, and 47.2 - 52.6 GHz[14]. Purely electrical approaches face problems in the generation of signals above 25 GHz, while their distribution is highly challenging due to the propagation losses of coaxial cables.

Microwave photonic approaches for the generation of mm-wave signal leverage the main advantages of optics in terms of broadband operation and low propagation losses, coupled with a high degree of reconfigurability if a programmable photonic processor is employed.

Among the existing techniques used for optically generating mm-Wave signals, one of the most widely used approaches consists of employing an external modulator and takes advantage of its frequency up-conversion inherent capability, when a continuous wave signal provided by a laser is modulated by an RF tone. Then, a proper configuration of the EOMs, in conjunction with filtering elements, can be exploited to select high-order modulating sidebands at their output and carry out the input signal frequency multiplication after the photodetection stage[15-17].

The schematic of the experimental setup employed for the generation of mm-wave signals by frequency multiplication using our programmable processor is shown in Supplementary Fig. 28.

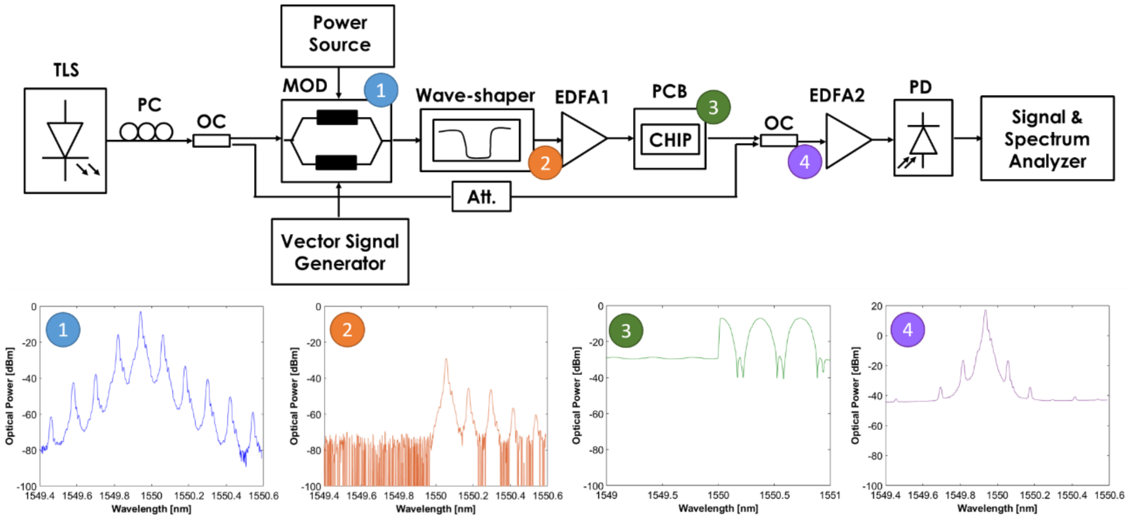

Supplementary Fig. 28: **Experimental setup used for the optical generation of mm-Wave signals and the measured responses of the signals at several points of the setup:** (1) Full-modulated signal. (2) Modulated signal after the wave-shaper, which removed the lower side bands and the optical carrier. (3) Amplified optical filter used to select the desired harmonic tone. (4) Optically modulated signal after carrier reinjection.

The light from a tunable laser is split into two paths through a 1x2 optical coupler (OC). On one path, the light is delivered to an intensity modulator (MOD, Sumitomo T.DEH1.5-40-ADC) driven by a signal at the RF input frequency signal to be multiplied. In this experiment, we used an RF signal at f_{RF}=15GHz. A tunable optical filter (wave-shaper 4000 form Finisar) is then used to remove both the optical carrier and the lower sidebands of the modulators. After an amplification stage, an optical integrated filter is synthesized in the photonic processor, to select a single tone and ensure the suppression of all the remaining spectral components. Finally, a

second optical coupler (OC) recombines the isolated tone with the optical carrier coming from the second output of the input OC, and the resulting signal is optically amplified and delivered to a high-speed PD (XPD3120R Finisar) at the circuit output. At the PD output, an RF carrier wave is generated at the beating frequency given by the detuning between the optical carrier and the selected modulator harmonic. Tuning the synthesized filter in the mesh by sweeping the cross phases of some PUCs in the filter path for selecting different tones allows synthesizing RF signals at integer multiples of the original RF frequency (N x 15GHz).

The optical response of the filter synthesized in the mesh and employed to select the desired harmonic tone is shown in Supplementary Fig. 29 and properly tuned to select the tone of interest by setting the cross-phase shifters of the PUCs involved in the filter path. As can be noticed from the figure, when the second harmonic (HD2) at 30GHz is selected, an optical rejection respect to the third harmonic (HD3) at 45GHz up to 12dB is measured and a rejection respect to HD2 of almost 15dB is obtained in the opposite case, that is, when the HD3 is selected.

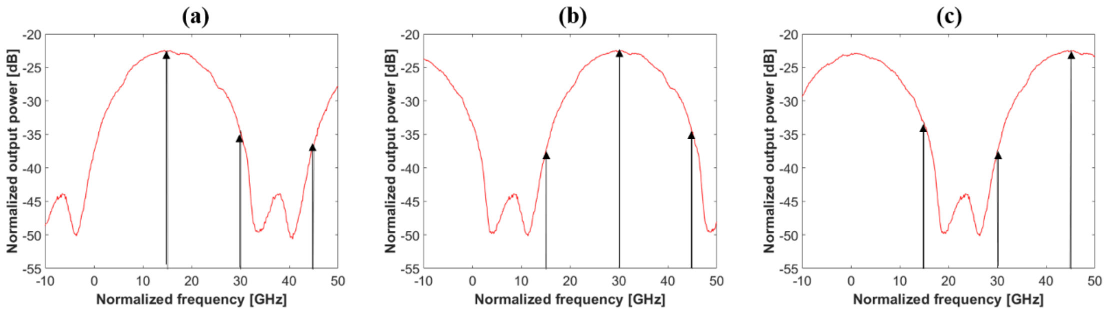

Supplementary Fig. 29 **Optical response of the synthesized filter in the mesh to select the desired harmonic tone when the cross-phases are tuned for selecting** (a) the fundamental tone (f_{RF}=15GHz), (b) the second harmonic (f_{HD2}=30GHz), and (c) the third harmonic (f_{HD3}=45GHz).

We carried out a continuous sweeping of the cross-phases while evaluating the optical modulated signal after the carrier reinjection and before the photodetection stage. Results are shown in Supplementary Fig. 30. In the center of the figure, the mapped optical power as a function of the cross-phase for the three involved signals (fundamental, HD2, and HD3) besides the optical carrier is presented, and the resulting shape in all cases is the optical response shape except for the optical carrier, as expected. This figure allows us to choose the proper cross-phase for selecting the desired harmonic tone, with a maximum rejection of the other ones. In both sides of the figure, some examples of the optical spectra of the modulated signals for several cross-phases are shown.

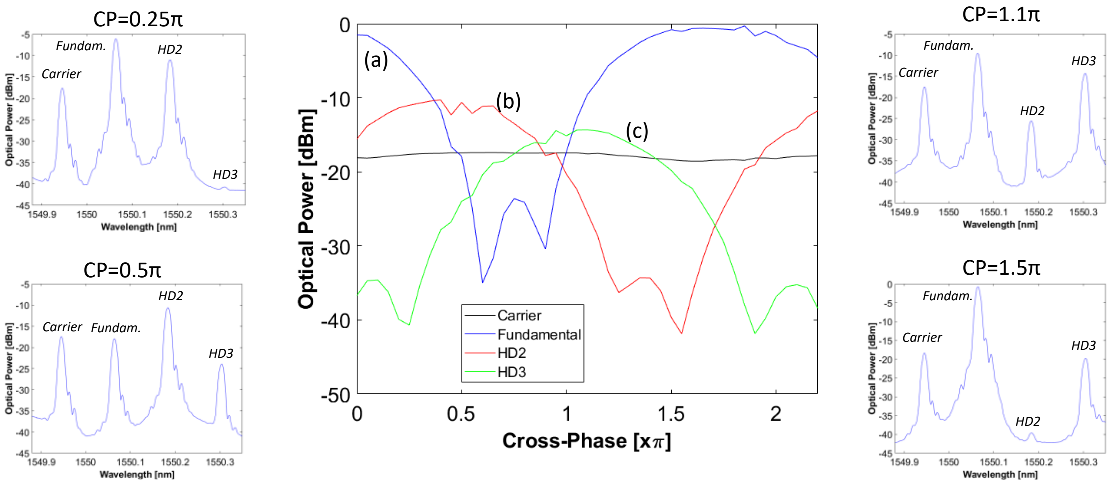

Supplementary Fig. 30 **Optical response of the synthesized filter in the mesh to select the desired harmonic tone when the cross-phases are tuned for selecting** (a) the fundamental tone (f_{RF}=15GHz), (b) the second harmonic (f_{HD2}=30GHz) and (c) the third harmonic (f_{HD3}=45GHz). The surrounding insets show the power of the optical carrier, fundamental tone and second/third order distortion tones for different settings of the cross-phase (CP) parameter.

Once the cross-phase shifter values are selected for HD2 and HD3 isolations, the modulated signal is photodetected and measured in the RF domain by a signal and spectrum analyzer. The resulting RF signals are presented in Supplementary Fig. 31 corresponding to different frequency multiplication factors (x2 and x3). We obtained a 22 dB (Supplementary Fig. 31 (a)) and 19,5 dB rejection (Supplementary Fig. 31 (c)) respectively for the cases of HD2 and HD3 selections for the other harmonics. Supplementary Fig. 31 (b) and Supplementary Fig. 31 (c) provide a zoom of the selected harmonics (HD2 and HD3 respectively) demonstrating a high spectral purity of the generated mm Wave signal.

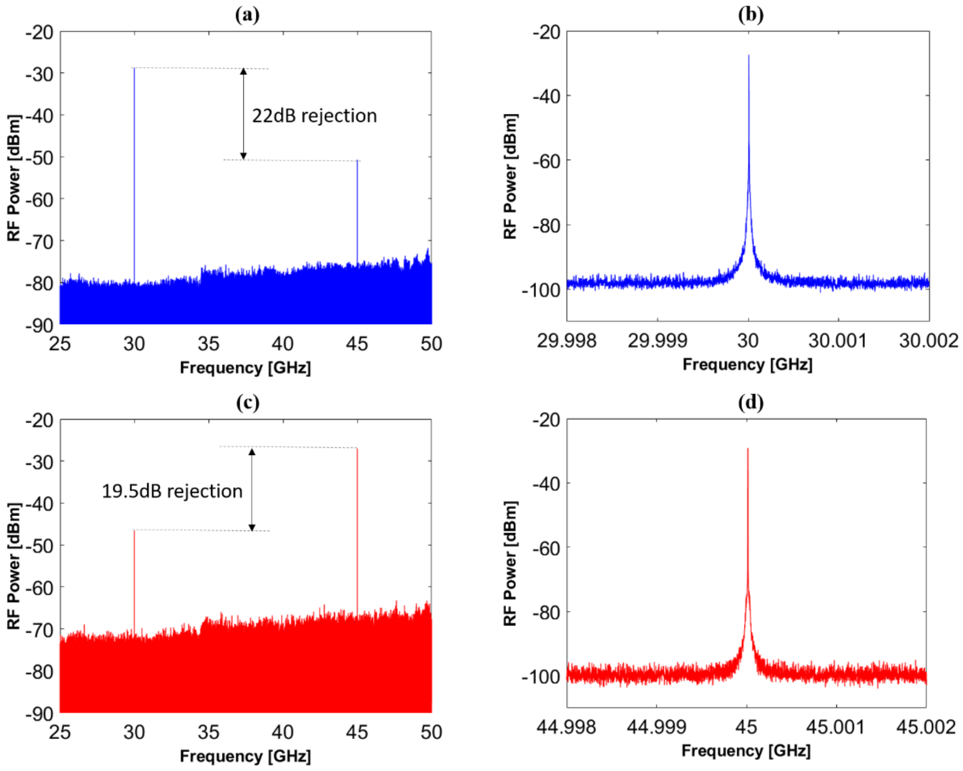

Supplementary Fig. 31. **Measured photodetected signal spectra** (a) when HD2 is selected by the filter, (b) zoom of the HD2 signal, (c) when HD3 is selected by the optical filter and (d) zoom of the HD3 signal.

Finally, we measured the phase noise (PN) for the generated mm-wave tones. PN requirements become more and more strict as the spacing between carriers is reduced, for example for high-bit-rate 5G applications[18]. Supplementary Fig. 32 shows the curves of the measured power spectral density (PSD) of the phase noise for the different multiplication factors (N=2 and N=3). Moreover, the comparison between these PN PSD curves measured for different frequency multiplication factors, N, and the RF input signal (electrical back-to-back), and the modulator and photodetector link, is also displayed. As shown from the plot, the measured PN PSD in [dBc/Hz] increases with the multiplication factor N as 20log10(N), following the theory. For instance, the PN PSD at 30GHz (i.e., N= 2) and 45GHz (i.e., N= 3) shows a 3dB difference.

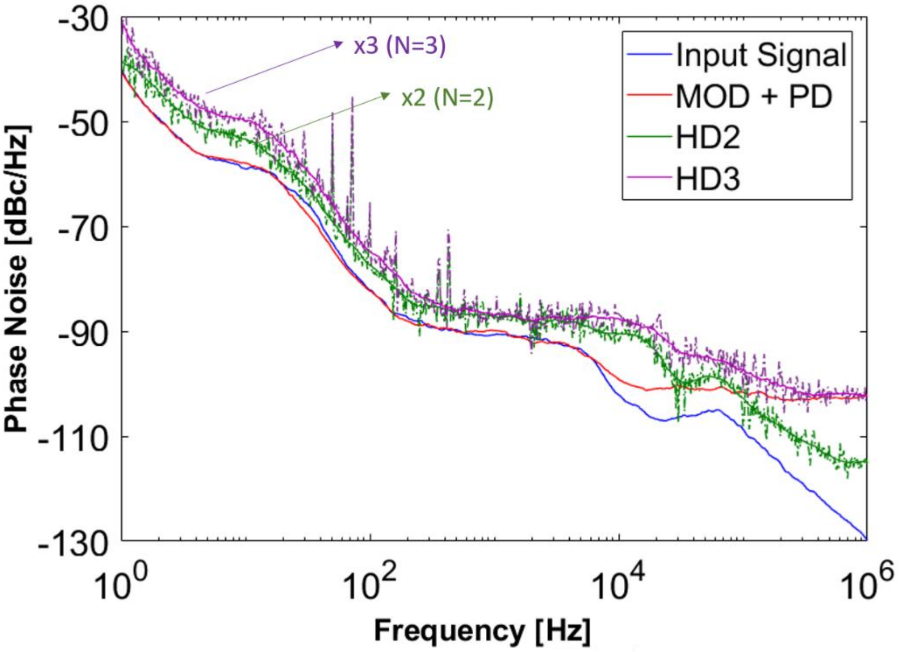

Supplementary Fig. 32 **Measured power spectral density of the phase noise for the RF input signal (electrical back-to-back), the modulator and photodetector link response, and the mmWave generated signals at HD2 and HD3 frequencies.**

Supplementary Note 7.

Arbitrary RF waveform generation

This application targets the generation of ultra-broad bandwidth RF waves with arbitrary and reconfigurable phase or amplitude characteristics. These are required in applications such as pulsed radar, medical imaging, modern instrumentation systems, and ultra-wideband (UWB) communications. Again, since the electronic approach is limited in frequency and bandwidth to a few GHz a solution is to take advantage of the high speed and broadband characteristic offered by photonic techniques and generate the microwave arbitrary waveform in the optical domain. They typically involve the generation of delayed and scaled replicas of RF signals or impulses in the optical domain with subsequent recombination and scaling to achieve the desired RF output[19].

In this experiment, we program reconfigurable UMZI lattice filters of different order as an on-chip pulse shaper and use them to generate replicas of RF impulses at eight different delays using an ultra-fast optical pulse source as a seed input. Pulses are scaled in amplitude by synthesizing the coupling factors in the photonic mesh to specified amplitudes before recombination. This technique is based on direct time-domain synthesis and eliminates the requirement of a highly dispersive and physically long medium. For that, we first programmed UMZIs lattice filters (of a different order, see Supplementary Note 3) with arm length differences of 2BULs and 4BULs obtaining different delays and therefore different RF central frequencies in each case.

Supplementary Fig. 33 depicts the experimental setup employed to carry out the time-domain RF waveform synthesis, where a short-pulse laser is tailored by a multi-stage (four in our case) integrated pulse shaper in both the frequency and time domains and is converted to an RF waveform using a photodetector after an amplification stage.

As mentioned in the main manuscript, the total Basic Unit Length (BUL) of the PUCs in the mesh is 811µm and the group index is 4.18, resulting in a Basic Unit Delay (BUD) of 11.25ps approximately (see Supplementary Note 2 for experimental verification of this figure). This defines the minimum time discretization to synthesize the interferometric structures. In our case, for the lattice filters with ΔL=2BULs and ΔL=4BULs, the achieved delay between replicas will be ΔT=22.5ps and ΔT=45ps, respectively.

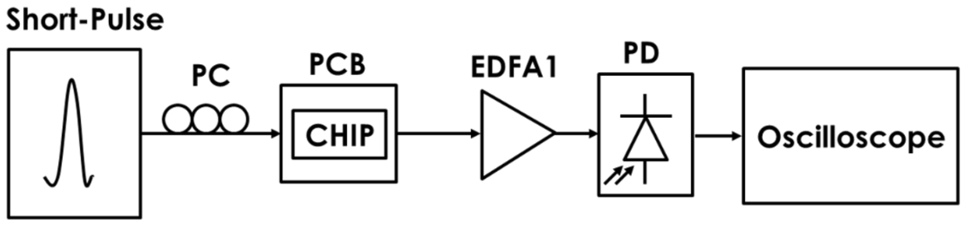

Supplementary Fig. 33: **Experimental setup used for arbitrary waveform generation**.

For the experiment, we used a high-speed pulsed fiber laser (from Calmar Optcom) emitting picosecond short pulses. Supplementary Fig. 34 shows the characteristics of this source measured both in the optical domain as a function of the wavelength (Supplementary Fig. 34 (a)) and in the time domain measured with an oscilloscope (Supplementary Fig. 34 (b)) and with an auto-correlator ((Supplementary Fig. 34 (c)). As it can be observed in the oscilloscope response, the pulse frequency repetition rate is 5GHz, which, in time, corresponds to an inter-

seed pulse delay of 200ps. This sets the maximum number of replicas to avoid overlapping. For example, in the case of ΔT=22.5ps, this number is 8 while for the case of ΔT=45ps, the number is 4. Moreover, as obtained from the auto-correlator response, the full width at half maximum of the generated short pulse is around 8.8ps. This means that the pulse width is around 4.4ps < 11.25 ps.

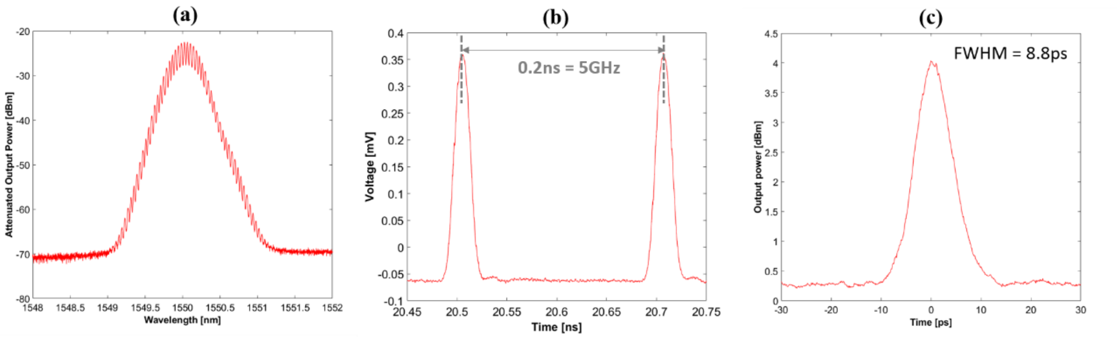

Supplementary Fig. 34: **Spectra and time domain responses for the short pulse source used in the experiments.** (a) Optical spectrum emitted by the pulsed laser measured in an optical spectrum analyzer. (b) Time domain short pulse train measured in an oscilloscope. (c) Time domain short pulse measured in an auto-correlator.

We first programmed cascade UMZI lattice filters of orders 1 to 4 with an arm unbalance of 2BUL. As shown in Supplementary Fig. 35 (a)-(d) respectively.

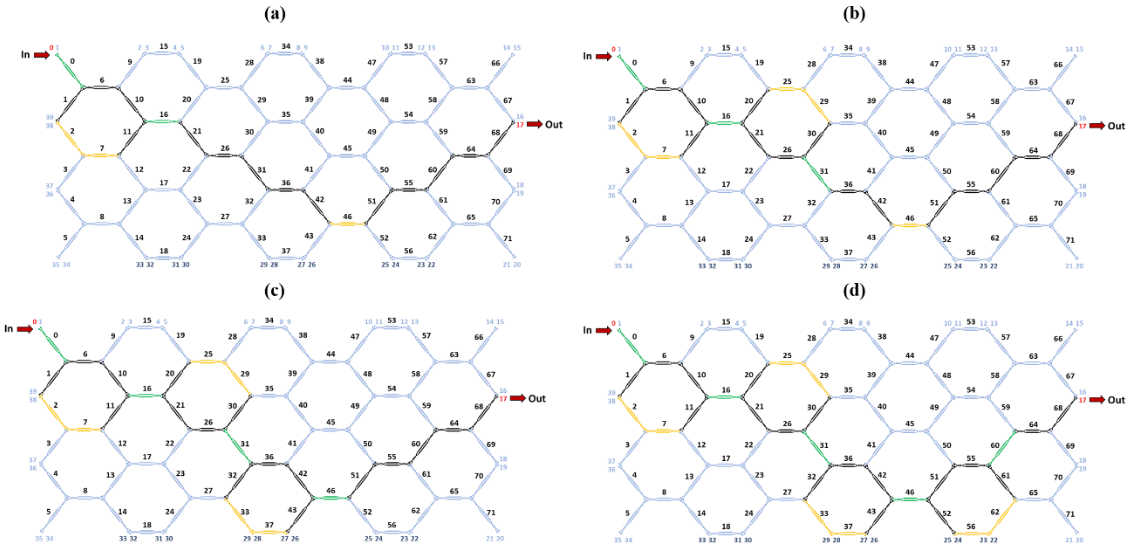

Supplementary Fig. 35: **Waveguide mesh connection diagram for the lattice cascaded UMZI (2BUL arm unbalance) filt**er (a) 1st order, (b) 2nd order, (c) 3rd order, and (d) 4th order. (Green: Tunable Coupler, Black: Cross State, Ochre: Bar state)

The processor was subsequently fed with the seed pulse and the results were recorded for different settings of the filter parameters to synthesize different waveforms. The main results are shown in Supplementary Fig. 36. The number of replicas is given in each case by the filter order +1, so order 1 lattice UMZI gives 2 replicas per seed pulse, order 2 lattice UMZI filter gives 3 replicas per seed pulse, and so on. Time separation between consecutive pulses is around 23 ps which implies an RF central frequency for the resulting wave packet of 43.5 GHz. As it can be observed, with higher-order filters it is possible to synthesize a wider number of wave packet forms. It is interesting to note that a higher operation frequency of 87GHz can be obtained if

instead of using the lattice UMZI filter we resort to other architectures of transversal filters allowing for ΔL=1BUL, ΔT=11,25 ps. We do not show these results as our oscilloscope could not provide enough bandwidth for this particular case. Note as well that although up to 8 replicas are possible, we report a maximum of 5. The reason for this is that the photonic waveguide mesh of the processor does not have enough PUCs to implement a seventh-order UMZI lattice filter.

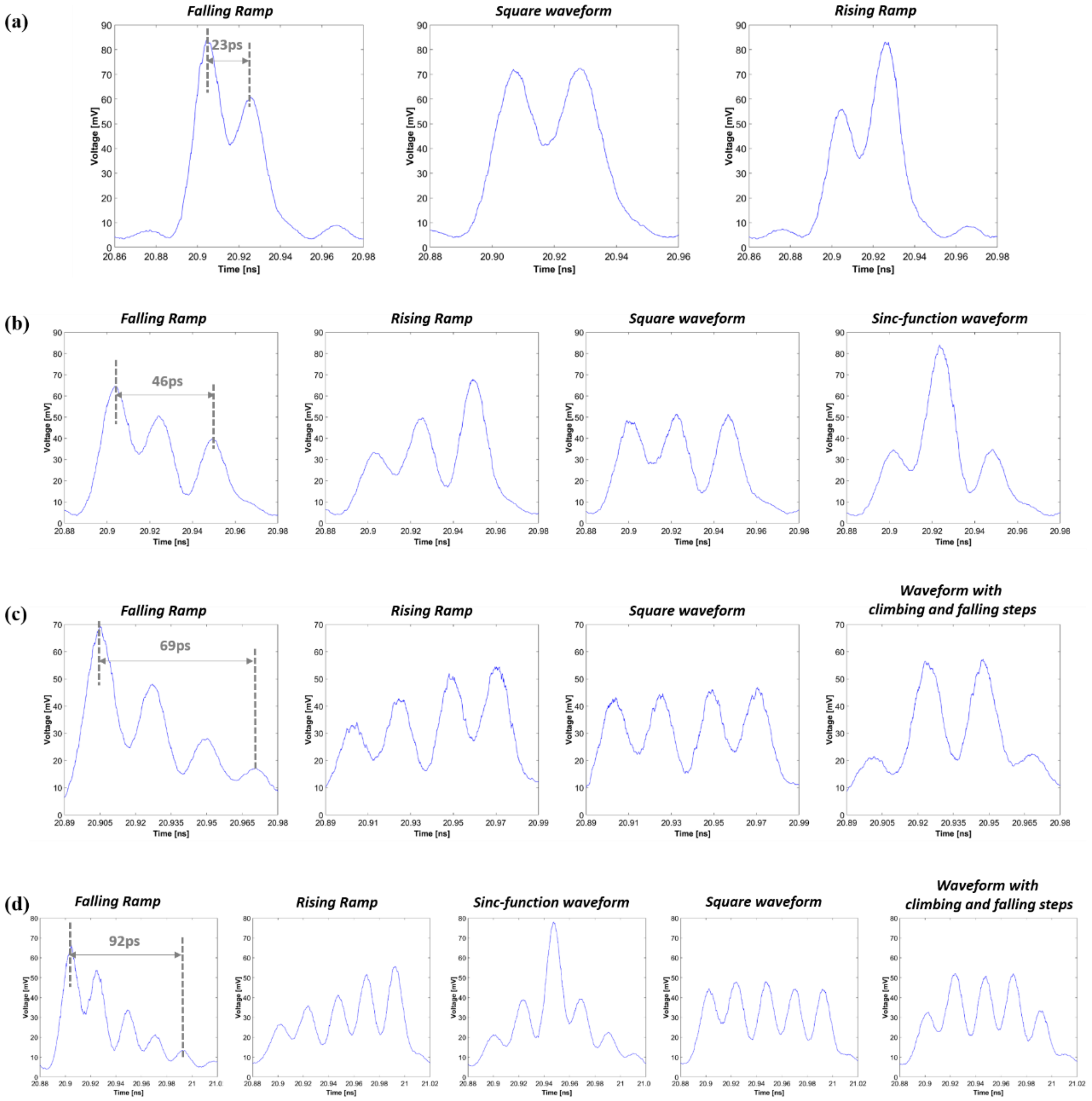

Supplementary Fig. 36: **Synthesized arbitrary waveforms using the programmable processor implementing waveguide mesh connection diagram for the lattice cascaded UMZI (2BUL arm unbalance) filter** (a) 1st order, (b) 2nd order, (c) 3rd order, and (d) 4th order. (Green: Tunable Coupler, Black: Cross State, Ochre: Bar state)

In a second step, we programmed cascade UMZI lattice filters of order 1 to 3 with an arm imbalance of 4BUL. As shown in Supplementary Fig. 37 (a)-(c) respectively. The processor was then fed with the seed pulse and the results were recorded for different settings of the filter parameters in order to synthesize, again, different waveforms. The main results are shown in Supplementary Fig. 38. The number of replicas is given, as in the previous case by the filter order +1. Time separation between consecutive pulses is now 46 ps which implies an RF central frequency for the resulting wave packet of 21.75 GHz. One can synthesize finite impulse response filters with increasing unbalance (6, 8, 10,12 BULs) to synthesize RF waveforms with decreasing

central frequency (i.e. 14,5, 10,8, 8.7, and 7.25 GHz, etc..) spanning therefore different regions of the spectrum from the S to the W band.

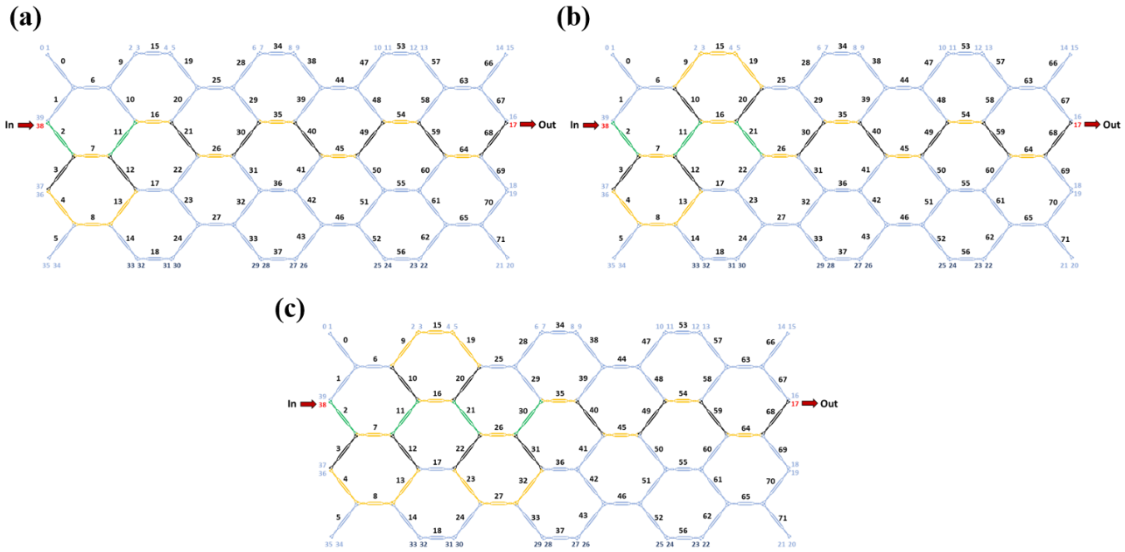

Supplementary Fig. 37: **Waveguide mesh connection diagram for the lattice cascaded UMZI (4BUL arm unbalance) filters** (a) 1st order, (b) 2nd order, (c) 3rd order (Green: Tunable Coupler, Black: Cross State, Ochre: Bar state)

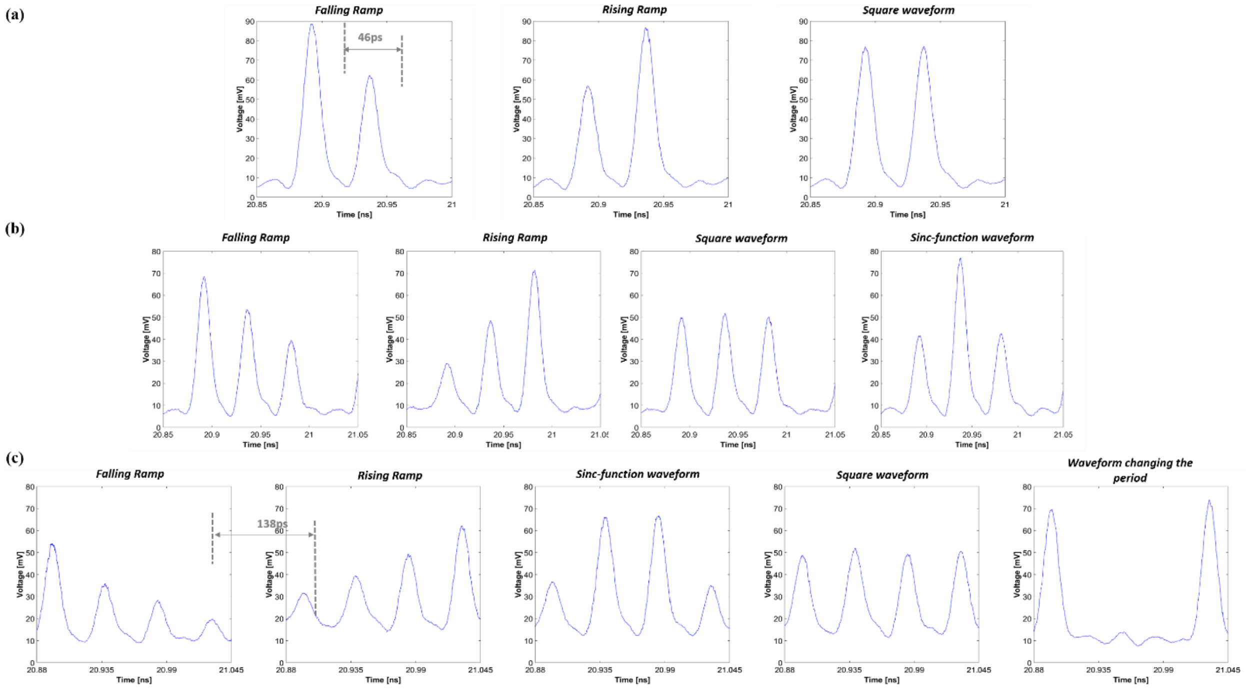

Supplementary Fig. 38: **Synthesised arbitrary waveforms using the programmable processor implementing waveguide mesh connection diagram for the lattice cascaded UMZI (4BUL arm unbalance) filter** (a) 1st order, (b) 2nd order, (c) 3rd order.

Supplementary Note 8.

Tuneable delay lines and beamforming

Microwave photonics technology has enabled the introduction of optical true-time delay line (OTTDL) technology into phased array PPAs avoiding the appearance of the beam squint effect and extending the operating bandwidths of PAAs. In particular, on-chip integrated microwave photonics beamformers based on switched delay lines can offer significant delays with wide bandwidth and low sensitivity to temperature fluctuations[20-23] at the expense of discrete tuning of the time delays.

One of the most relevant features of our photonic processor is that it can enable the programming of different true time delay lines between a selected set of input and output ports. Moreover, the fixed incremental delay ΔL=N.BUL (N=..-3-2,-1,0,1,2,3..) between output ports feeding adjacent antenna elements can be programmed to change thus enabling the reconfiguration of the beam pointing angle.

We programmed our processor to enable a four-element beamformer capable of pointing up to 9 angles (4 positive, broadside, and 4 negative in a -55º to 55º range. Supplementary Fig. 39 shows the waveguide mesh programming for the broadside (0º) and 4 positive angles (ΔL= 1 BUL→θ=13.7º, ΔL= 2 BUL→θ=27.4º, ΔL= 3 BUL→θ=41.25º, ΔL= 4 BUL→θ=54,9º). A similar reversed configuration provides the negative pointing angles.

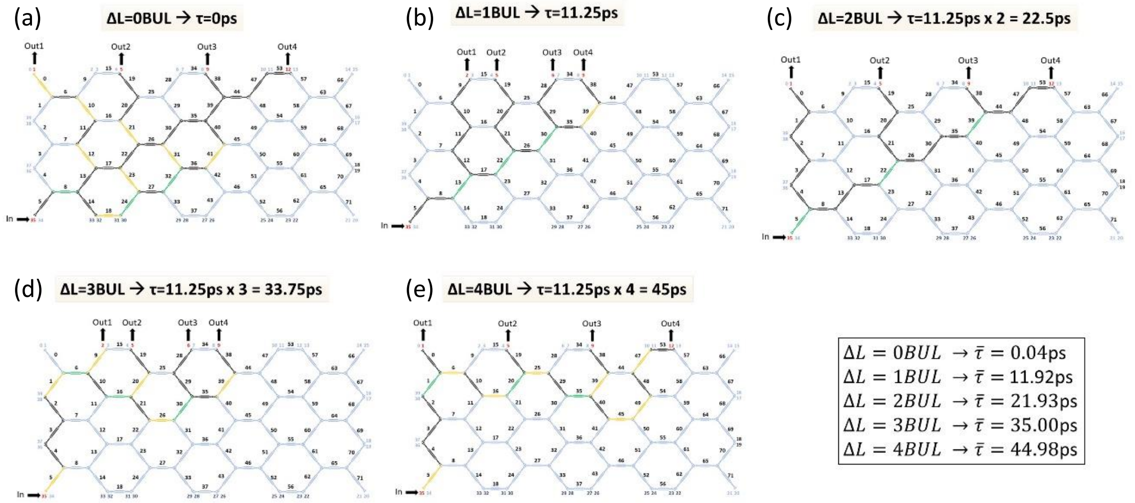

Supplementary Fig. 39: **Waveguide mesh programming in the photonic processor for the implementation of 4 delay lines with fixed incremental delays of 1 BUL.** (a) ΔL=0 BUL, (b) ΔL=1 BUL, (c) ΔL=2 BUL, (d) ΔL=3 BUL, (e) ΔL=4 BUL. (Green: Tunable Coupler, Black: Cross State, Ochre: Bar state)

The time delay performance of each configuration was measured using the experimental setup shown in Supplementary Fig. 40. The processor was fed with an input pulsed signal from a LUNA Optical Vector Analyzer OVA). The different beamformer configurations were programmed in the processor using the multichannel electronic driving and the signals from the different outputs were fed back to the analyser using the processor fiber array. The OVA provided the amplitude and delay characteristics of each input/output port pair.

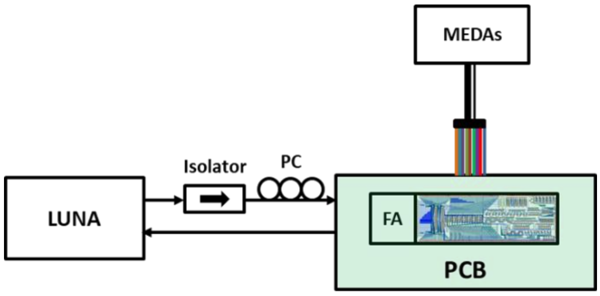

Supplementary Fig. 40: **Experimental setup used in the measurement of the programmable beamformer waveguide mesh programming**

The measured results for the broadside and positive beam pointing angles corresponding to the waveguide mesh configurations displayed in Supplementary Fig. 39 are shown in Supplementary Fig. 41 for a wavelength of 1550 nm. The measured values for the incremental delays in each configuration are shown in the table inset of Supplementary Fig. 39.

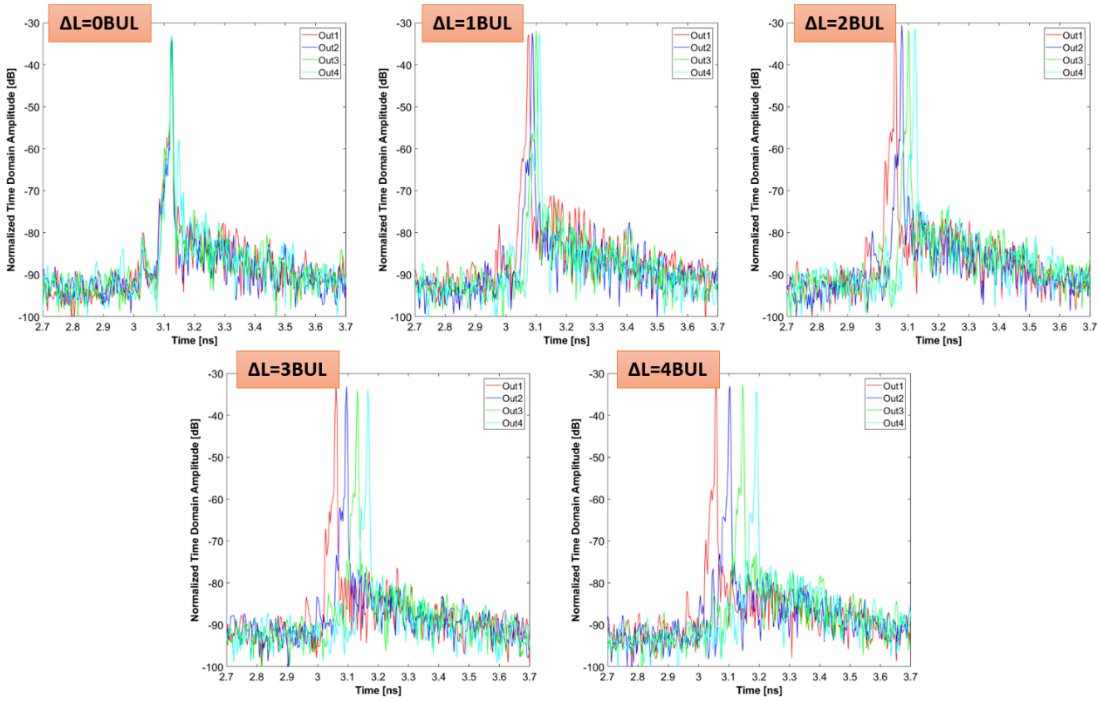

Supplementary Fig. 41**: Measured group delay responses for the 4 elements of the beamformer corresponding to the configurations for broadside and positive beam pointing angles of Supplementary Fig. 39**

Broadband operation was confirmed by measuring the delays in each configuration versus the normalized frequency range around 1550 nm. Results are shown in Supplementary Fig. 42.

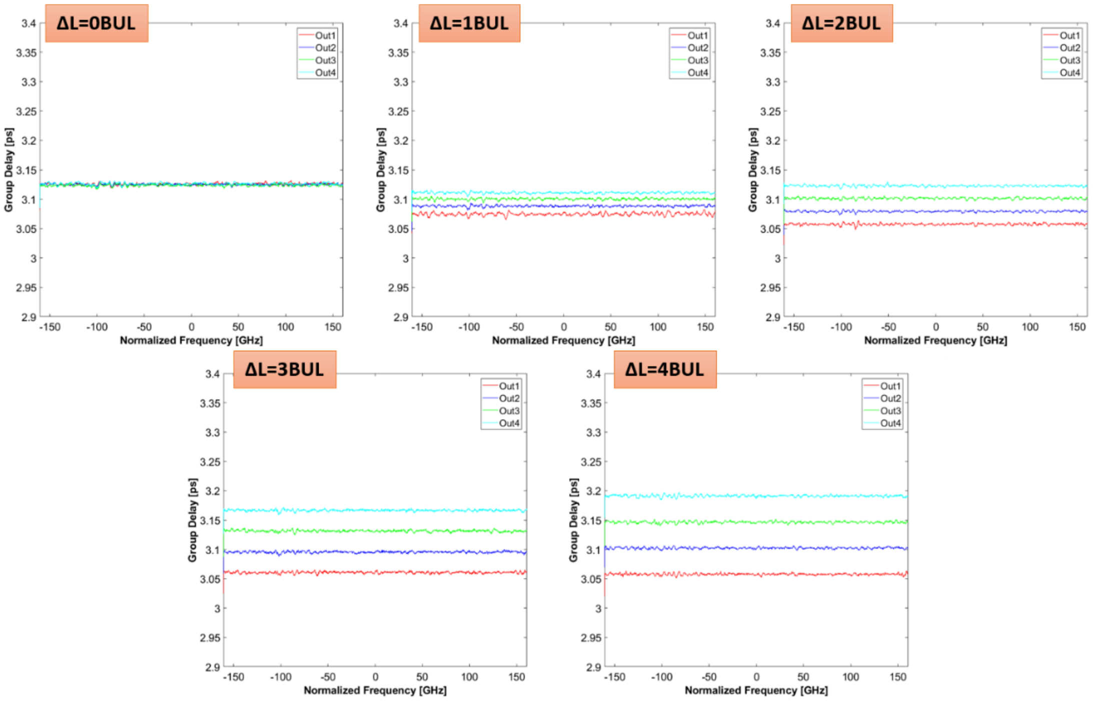

Supplementary Fig. 42: **Measured group delay responses versus the normalized frequency around 1550 nm for the 4 elements of the beamformer corresponding to the configurations for broadside and positive beam pointing angles of Supplementary Fig. 39.**

Broadband constant operation over 100GHz around the central frequency is observed. Finally, in Supplementary Fig. 43 we represent the beampattern corresponding to the experimental group delay results measured for positive, broadside and negative angles. The result shown in the figure corresponds to the array factor computed at 10 GHz operation frequency.

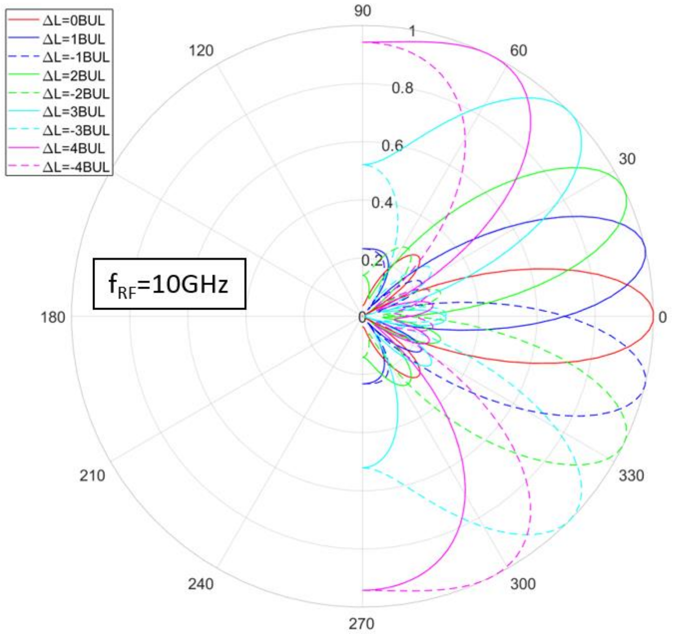

Supplementary Fig. 43: **Beampattern at 10 GHz corresponding to the experimental group delay results (Supplementary Fig. 42) measured for positive, broadside and negative angles.**

Supplementary Note 9.

5G signal interconnection and power division

We programmed our processor photonic mesh for the dynamic optical transmission, routing, and switching of digitally modulated radiofrequency signals. These operations enable more complex tasks like smart signal processing and management in data centers or radio-over fiber and 5G central offices[24-27]. For that purpose, on one hand, we programmed the mesh to perform optical signal interconnection between optical paths with different path lengths and on the other hand, a pair of reconfigurable optical splitters (1x2 and 1x4). Next, we introduced 5G standard signals to be transmitted through the mesh, that is, through the interconnects to evaluate the signal deterioration, and using the reconfigurable optical dividers, we demonstrated the flexible splitting capability of the mesh.

To measure the signal degradation, we employed the typical figure of merit to characterize MWP links, the error vector magnitude (EVM), which is measured at the receiver and gives a good indication of the signal quality. The EVM parameter is used in most wireless system standards such as Wi-Fi or in mobile networks such as 5G, to calculate the performance of a transmitter/modulator or receiver/demodulator implementing the respective standard. It relates the performance of the actual waveform compared to an ideal signal as calculated over the course of the ideal constellation. The lower the EVM after optical processing, the lower deterioration the signal will have suffered[28].

The setup used in this experiment is shown in Supplementary Fig. 44. A vector signal generator (SMW200A from Rhode&Schwarz) provides modulated signals that propagate through the optical chip processor before being photodetected and collected by an RF signal and spectrum analyzer (FSW43 from Rhode&Schwarz). The modulated signals are time division duplex (TDD) new radio (NR) 5G signals with 64 and 256-quadrature amplitude modulated (64-QAM and 256-QAM) modulation formats and 100 MHz bandwidth. Moreover, we used two frequencies within the two frequency ranges employed in the 5G NR standard[14]. The first one is the Frequency Range 1 (FR1) which includes a sub-6 GHz frequency band (f=5.9GHz) and the second is the Frequency Range 2 (FR2) including frequency bands in the mm-Wave range (f=26GHz and f=37GHz).

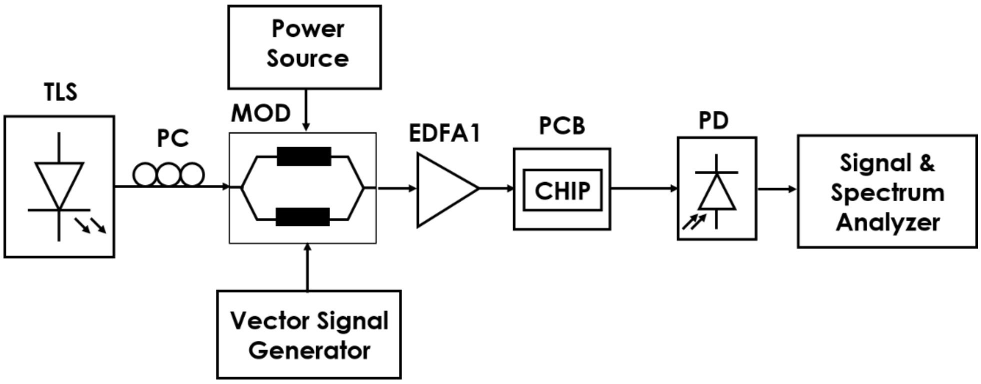

Supplementary Fig. 44: **Diagram of the experimental set-up used for the optical transmission of 5G digital signals.**

For the first part of the experiment, we reconfigured the mesh to implement interconnection paths with different lengths (from 8 to 20 PUCs). To evaluate the dispersion of EVM values, we

synthesized five paths of each length and measured the resulting EVM in the received signal for a fixed RF input power (for an optimum EVM in the longest path of 20BUL).

Supplementary Fig. 45 shows the received constellations of the 64-AQM (top) and the 256-QAM modulated signals (bottom) signals in the FR3 range, for 8-BUL length interconnects (left) and 20-BUL length interconnects (right). We can observe the degradation in the signal quality due to the increased length (i.e. losses) of the interconnections.

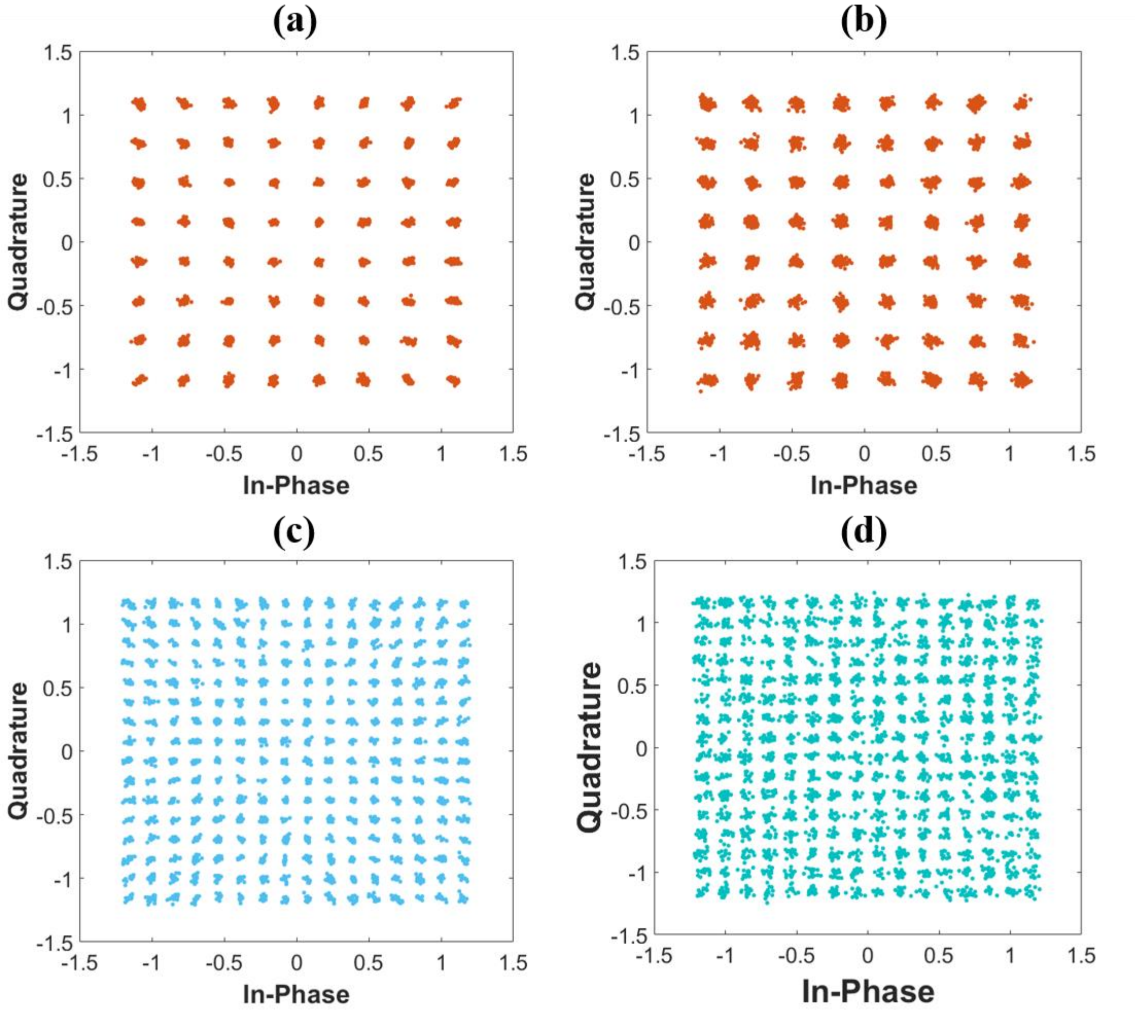

Supplementary Fig. 45: **Constellation diagrams corresponding to a frequency of 37GHz (FR2) 5G signal** (a) 64-QAM and 8BUL interconnect length, (b) 64-QAM and 20BUL interconnect length, (c) 256-QAM and 8BUL interconnect length and (d) 256-QAM and 20BUL interconnect length.

Supplementary Fig. 46 shows the measured EVM for each transmitted signal as a function of the number of PUCs crossed in the mesh for 64-QAM (Supplementary Fig. 46 (a)) and 256-QAM (Supplementary Fig. 46 (b))), at the three evaluated RF frequencies. As observed, there is a very low dispersion in the results obtained for paths of the same length. Moreover, both modulation constellations showed similar EVM, as expected. Predictably, the EVM is increased with the interconnection length, that is, with the increased number of PUCs (i.e losses) with a higher EVM obtained for higher transmission frequencies. Higher frequencies present a higher EVM degradation slope with the path length. The obtained EVM values are, in all cases, well below the allowed limit (3GPP TS 38.101-1 EVM requirements for different 5G modulation schemes: 8% for 64-QAM and 3.5% for 256-QAM[29]). This fact shows the high quality of the received signals

and therefore the potential of the photonic mesh as a system enabling smart signal processing, routing, and signal management of Radio-over-fiber signals.

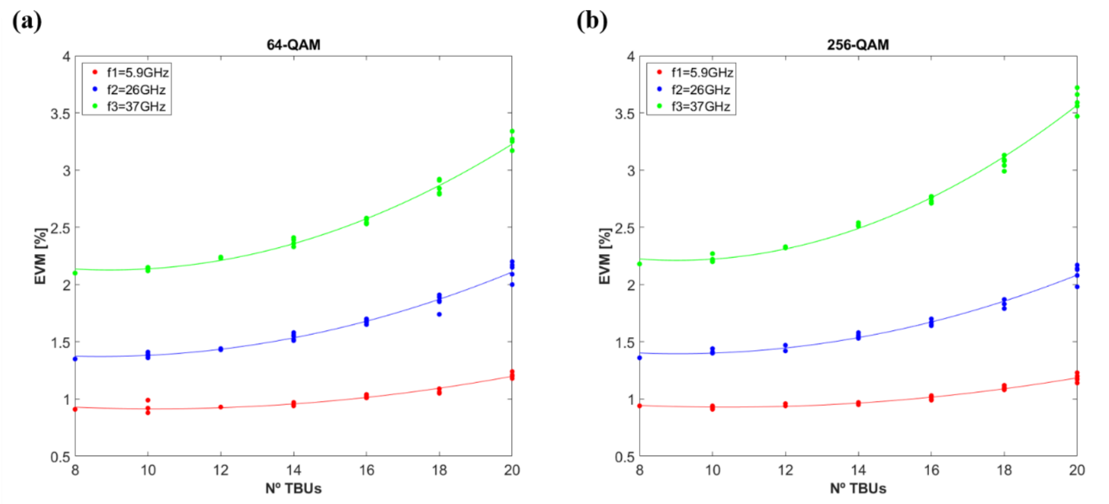

Supplementary Fig. 46: **Measured error vector magnitude as a function of the length of the path in the mesh in terms of the number of crossed unit cells or PUCs.** (a) 64-QAM and (b) 256-QAM modulated signals.

To figure out if signal degradation is due only to the optical losses associated with the PUCs themselves, or due to a non-optimum input RF power for each length path, we repeated the experiment but this time with a variable RF input power. Supplementary Fig. 47 (a) shows the results of the measured EVM for a fixed RF input power (for an optimum EVM in the longest 20BUL path) and the EVM for an optimum RF input power for each path length. As can be observed, received RF power strongly affects the EVM evolution. If the received RF power is optimized for each path length, a more linear behavior of the EVM with the path length is obtained. In addition, we confirm that this slope change is more significant for higher frequencies.

Finally, to evaluate the signal degradation due to the PUCs, we changed the position of the optical amplifier (EDFA) from before the chip to after the chip. Moreover, the previous experiments were made with a fixed EDFA gain, and now, a variable EDFA gain compensating the losses for each path length was employed. Results of the comparison of these two different configurations are presented in Supplementary Fig. 47 (b). The gain provided by the EDFA slightly flattens the EVM slope with the path length but not in a significant way, so we can conclude that PUCs are not the main cause of signal degradation apart from the optical coupling losses. Moreover, the EDFA after the chip, before the photodetector, and without a filter after it, produces higher noise, and therefore an increase of the EVM, as expected.

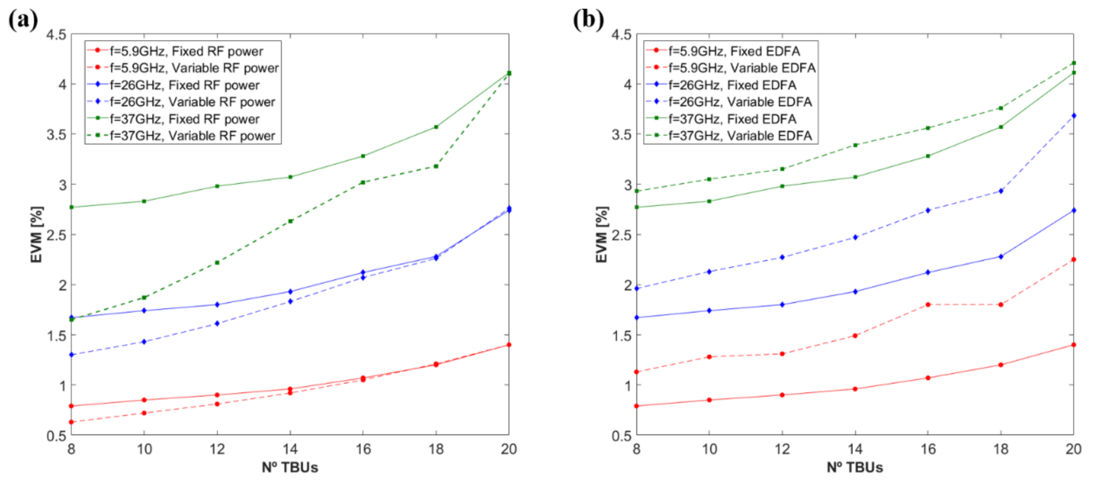

Supplementary Fig. 47: **Measured error vector magnitude as a function of the length of the path in the mesh in terms of the number of crossed unit cells or PUCs for 256-QAM modulated signals**. (a) Comparison of the RF input power fixed for all the path lengths and variable for each path length and (b) comparison of the EDFA before the chip and with a fixed gain and the EDFA after the chip and a variable gain (compensating the optical losses for each path length).

Our second experiment was to program the photonic processor as a reconfigurable power splitter and test it with the 5G signals. We employed the experimental setup of Figure S8.1 where the photonic processor was now programmed to operate as an even 1x2 and a 1x4 power divider. The measured results for the output signal in the case of a 26 GHz 64-QAM constellation are shown in Supplementary Fig. 48 and Supplementary Fig. 49 respectively. Excellent EVM values (1.68-1.69 for the 1x2 splitter and 2.12-2.14 for the 1x4 splitter were measured)

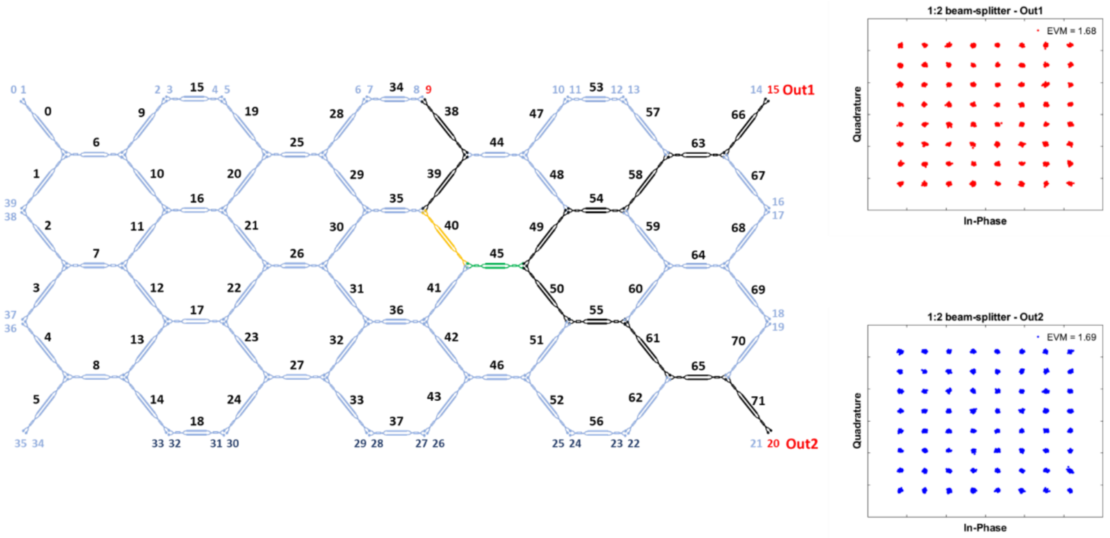

Supplementary Fig. 48: **1x2 Power divider programming for 5G QAM constellations:** Processor mesh programming and measured output constellations for a 1x2 power divider and a 5G 64-QAM constellation at 26 GHz.

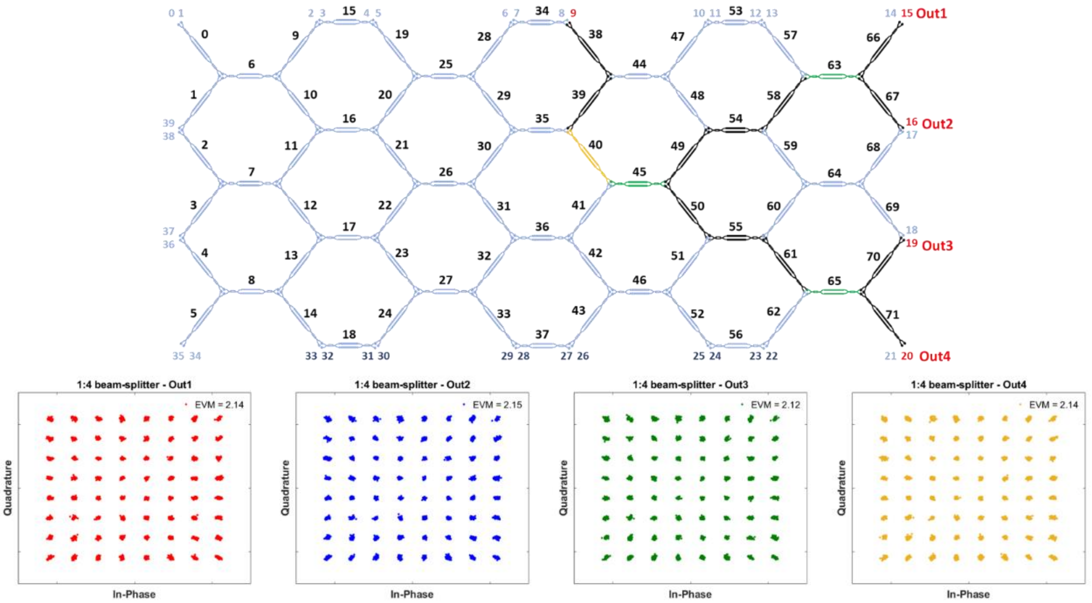

Supplementary Fig. 49: **1x4 Power divider programming for 5G QAM constellations:** Processor mesh programming and measured output constellations for a 1x4 power divider and a 5G 64-QAM constellation at 26 GHz.

Supplementary Note 10.

Switching, broadcasting and add-drop multiplexing

We programmed our photonic processor to demonstrate its capabilities to provide switching, broadcasting, and add-drop multiplexing for broadband and channelized signals. The first example, shown in Supplementary Fig. 50 corresponds to the switching and broadcasting of broadband signals. Supplementary Fig. 50 (a) provides the selected interconnection pattern, which includes two switching (interconnection) paths between two input and two output paths and two broadcasting connections between one input and two/four output ports. Supplementary Fig. 50 (b) provides the color diagram for the programmed waveguide mesh and finally, Supplementary Fig. 50 (c) displays the measured results.

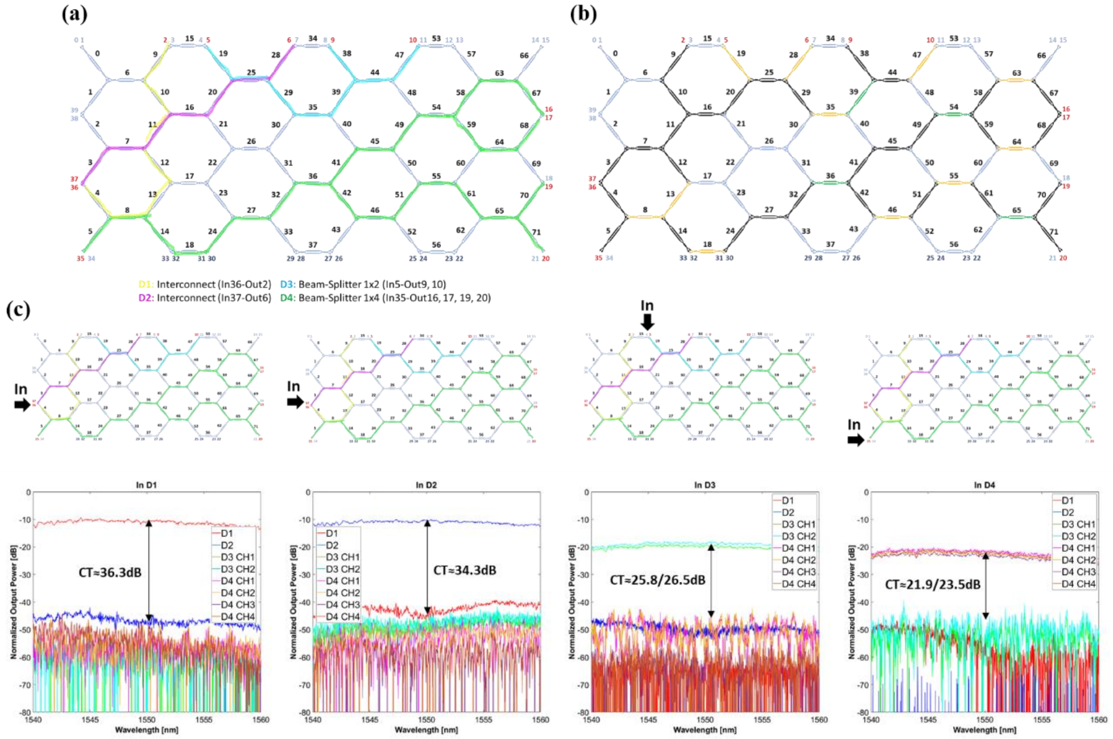

Supplementary Fig. 50: **Programmable broadband interconnection and broadcasting pattern examples:** (a) Interconnection pattern featuring two switching (interconnection) paths between two input and two output paths and two broadcasting connections between one input and two/four output ports. (b) color diagram for the programmed waveguide mesh (Green: Tunable Coupler, Black: Cross State, Ochre: Bar state, Blue: unused) (c) Measured results for each interconnection configuration D1-D3 including crosstalk for the rest of connections.

It should be noted that the measured results were taken with all the connections being simultaneously active and include, for each connection, the effect of undesired crosstalk from the rest. An excellent rejection level of over 33 dB is obtained for switched paths, while broadcast signals provide a minimum rejection close to 22 dB. With larger waveguide meshes and further optimization of routing paths, it is expected that these already remarkable results can be further improved. Note that a 40 nm operation range is obtained.

A second example shows the capability to provide simultaneous broadband and narrowband (i.e add-drop multiplexing and filtered) connection capabilities. In this case, broadband switched and broadcast connections are implemented together with an add-drop and a narrowband switched

connection as shown in Supplementary Fig. 51. Crosstalk rejection ratios are similar to the previous case for broadband connections, while for narrowband connections improve in the case of the filtered switching connection (>50 dB) and worsen for the case of the add-drop (20 dB for the add and 15 dB for the drop ports). In this case, the add-drop configuration was implemented by means of an unoptimized ring cavity yielding a poor drop response.

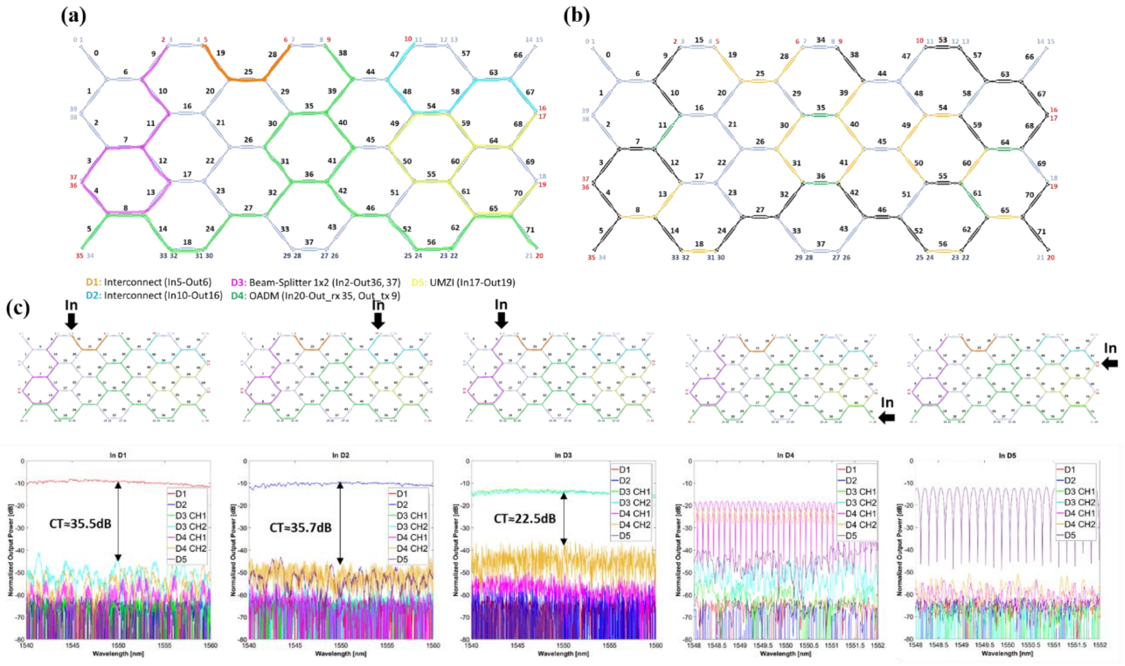

Supplementary Fig. 51: : **Programmable broadband and filtered switching and broadcasting pattern examples:** (a) Interconnection pattern featuring two switching (interconnection) paths between two input and two output paths, one broadcasting connection between one input and two output ports, one add-drop multiplexing connection between one input and two output ports, and one narrowband switching interconnection between one input and one output port. (b) color diagram for the programmed waveguide mesh (Green: Tunable Coupler, Black: Cross State, Ochre: Bar state, Blue: unused) (c) Measured results for each interconnection configuration D1-D3 including crosstalk for the rest of connections.

Supplementary Note 11.

Tunable frequency measurement

To implement a frequency measurement system we need to access the two complementary outputs of a 1x2 optical filter[30]. Ideally, the filter spectral period should be compatible with the frequency range to be measured, so the wider the better. This means that a MZI filter with the shortest path imbalance (2 BUL) is, in principle, the preferred option. We therefore programmed our processor to implement such a filter. Supplementary Fig. 52 (a) provides the color diagram for the programmed waveguide mesh, while Supplementary Fig. 52 (b) depicts the complementary (Cross & Bar) transfer functions for different values of the MZI coupling constants (k=0.5 to 0.58, with the highest suppression given by k=0.58).

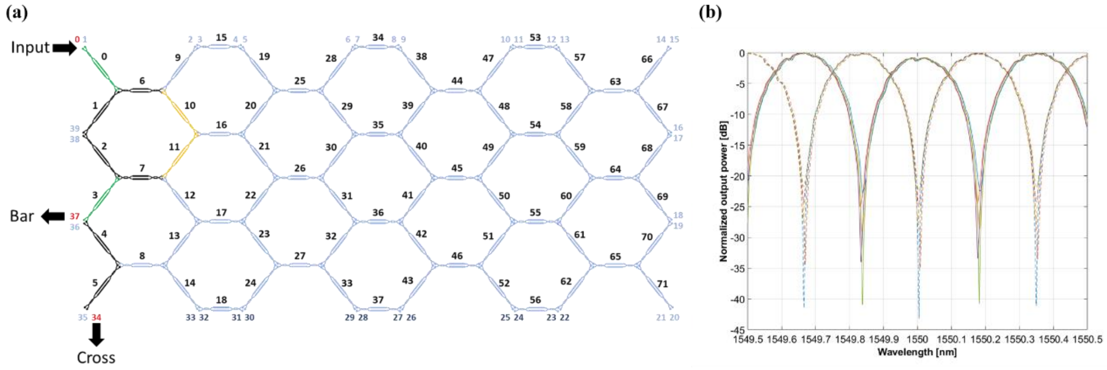

Supplementary Fig. 52: **Examples of ACFs for frequency measurement applications I:** Measured (a) Color diagram for the programmed waveguide mesh used to implement a 1x2 MZI Filter with path imbalance given by 2BUL (Green: Tunable Coupler, Black: Cross State, Ochre: Bar state, Blue: unused) (b) Measured results for the 1x2 MZI filter showing Bar (even maxima) and Cross (odd maxima) transfer functions and different values of the MZI coupling constants ranging from 0.5 to 0.58.

The frequency measurement system performance is given by the Amplitude Comparison Function *ACF(f)*, which is defined by[30]:

$$ACF(f) = \frac{H_{Cross}(f)}{H_{Bar}(f)} \quad (1)$$

In Supplementary Fig. 53 we show the measured results for the normalized *ACF(f)* for the transfer functions measured in Figure S10.(b) and zero extra phase shift between the arms.

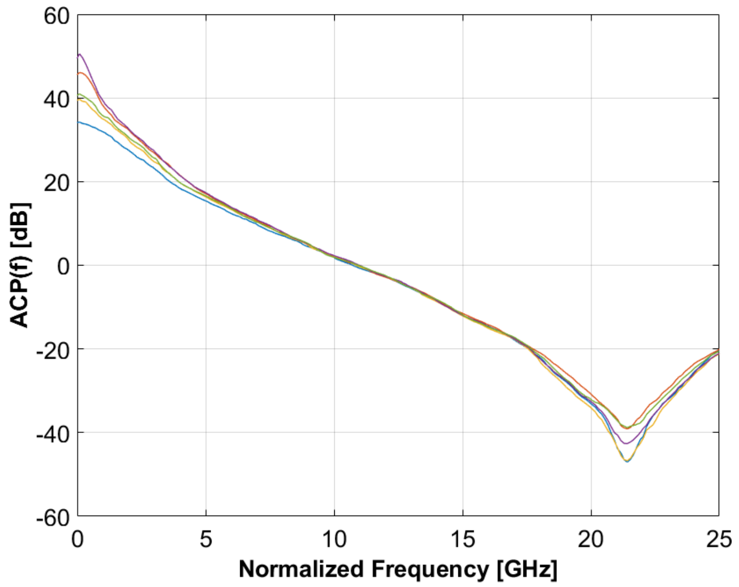

Supplementary Fig. 53: **Examples of ACFs for frequency measurement applications II:** Measured ACF(f) for the transfer functions displayed in Supplementary Figure 52(b) and zero extra phase shift between the arms

The ACF shows a linear performance within a spectral region of more than 12 GHz with a sensitivity of 2.75dB/GHz for this particular case. Note however that since the waveguide mesh is programmable, the extra phase shift between the rms can be changed resulting in different slope values and signs. This is shown, for instance in Supplementary Fig. 54, where we display the Cross and Bar transfer functions for different values of the extra phase shift (CP) between the MZI arms in Supplementary Fig. 54 (a) and the resulting ACFs in Figure Supplementary Fig. 54 (b). Note that both the slope value in dB/GHz can be changed as well as the positive and/or negative sign of it.

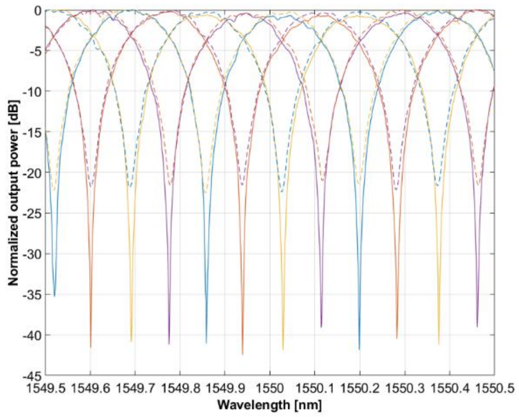

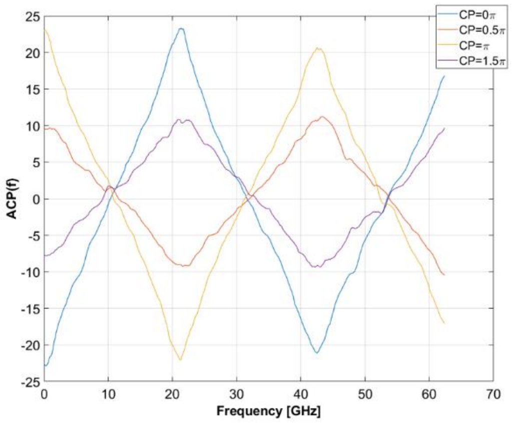

Supplementary Fig. 54: **Examples of ACFs for frequency measurement applications III:** (a) Cross and Bar transfer functions for different values of the extra phase shift (CP) between the MZI arms (K=0.58) (b) Resulting ACFs for each case (multiple periods are shown)

Supplementary Note 12.

Implementation of Optoelectronic oscillators

We programmed our photonic processor to demonstrate its capabilities to implement optoelectronic oscillators with reconfigurable capabilities. In the first experiment, we exploit internal cavity switching to change from a short to a long cavity device[31]. In the second experiment, we programmed the same processor to implement via a power division operation a dual loop Vernier OEO[32] and increase the overall cavity-free Spectral Range.

Supplementary Fig. 55 shows the layout for the first experiment, where two cavities (paths 1 & 2) are switched using our programmable processor as shown in the upper part of the Figure. The short cavity OEO (path 1) features an optical path of 53m and an electronic path of 1.35 m (overall spectral period around 3.1 MHz). The long cavity OEO (path 2) features an optical path of 1.4 Km and an electronic path of 1.35 m (overall spectral period around 1.38 kHz). In each case we measured the OEO spectrum around 1.5 GHz and 10 GHz by inserting a 50 and a 430 MHZ filter respectively centred at those frequencies. For the measurements, we used a IQS-2403BLD-XX-P7-EA EXFO laser, a Sumitomo T.DEH1.5-40-ADC modulator, three optical amplifiers (EDFA1: Amonics C-BAND AEDFA-23-B-FA and EDFA2/EDFA 3: Amonics C-BAND AEDFA-27-B-FA) a Finisar XPD3120R photodiode an Agilent, Signal Analyzer MXA, N9020A, and a SHF M804 B RF amplifier.

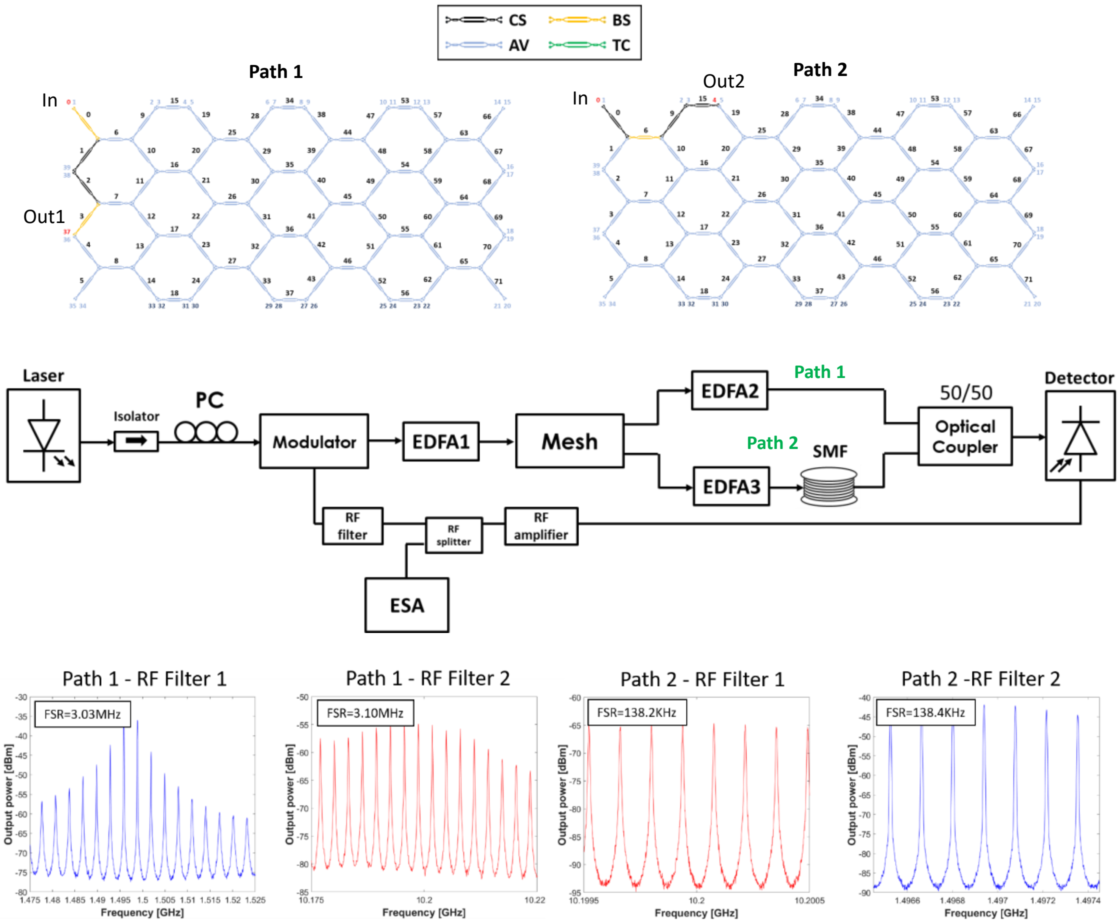

Supplementary Fig. 55: **Layout of the experimental configuration assembled to demonstrate a switched OEO configuration by suitable programming of the RF-Photonic processor**. The upper part shows the internal programming of the waveguide mesh to implement the short (path 1) and the long (path 2) OEOs.

The intermediate part shows the experimental setup and the lower part shows the spectra of both OEOs around 1.5 and 10 GHz.

The lower part of Supplementary Fig. 55 shows the measured results for the short (path 1) and long (path 2) OEOs centered at 1.5 GHz (RF filter 1) and 10 GHz (RF filter 2). The oscillations are clearly visible with tone tone-to-floor ratios in the 30 to 40 dB. Note the change in the free spectral range as the short cavity is switched to the long.

Supplementary Fig. 56 shows the layout for the second experiment, where a dual loop OEO composed of two very similar cavities (paths 1 & 2) are combined using our programmable processor as shown in the upper part of the Figure. The first cavity (path 1) features an optical path of 53m and an electronic path of 1.2m (overall spectral period around 3.1 MHz). The second cavity (path 2) features an optical path of 73m and an electronic path of 1.2m (overall spectral period around 2.31 MHz).

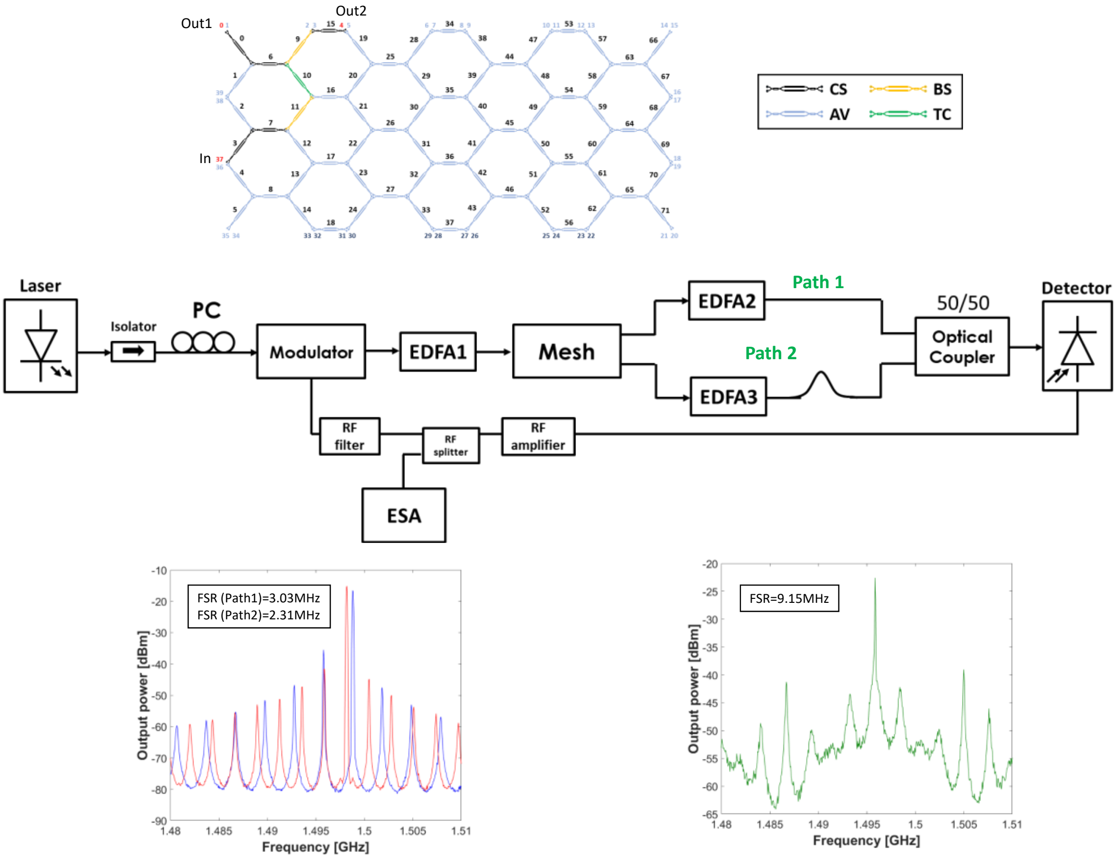

Supplementary Fig. 56: **Layout of the experimental configuration assembled to demonstrate a dual loop Vernier OEO configuration by suitable programming of the RF-Photonic processor.** The upper part shows the internal programming of the waveguide mesh to implement as a 1x2 power divider. The intermediate part shows the experimental setup and the lower part shows the spectra around 1.5 GHz of both OEO cavities before combination(left) and after combination (right).

The spectral response of each separate cavity around 1.5 GHz is shown in the lower left part of Figure S11.2. The spectral periods are 3.03 and 2.31 MHz respectively. The spectral response of the combined dual-loop cavity is shown in the lower right part. The spectral period is now 9.15 MHz, which matches the theoretical value predicted by the Vernier formula[32]:

$$FSR_{total} = \frac{FSR_1 . FSR_2}{|FSR_1 - FSR_2|} \approx 9.6\ MHz \tag{2}$$

References

1. López-Hernández, A., Gutiérrez-Zubillaga, M. & Pérez-López, D. Automatic Self-calibration of Programmable Photonic Processors, IEEE Photonics Conference (IPC), Vancouver, BC, Canada, doi: 10.1109/IPC53466.2022.9975587 (2022).

2. López-Hernández, A., Pérez-López, D., DasMahapatra, P. &Capmany, J. Auto-routing algorithm for field-programmable photonic gate arrays. *Opt. Express* **28**, 737-752 (2020).

3 Pérez-López, D., López-Hernández, A., DasMahapatra, P. & Capmany, J. Multipurpose self-configuration of programmable photonic circuits. *Nat. Commun.*, **11**(1), 6359 (2020).

4 Pérez-López, D. Programmable Integrated Silicon Photonics Waveguide Meshes: Optimized Designs and Control Algorithms. *IEEE J. Sel. Top. Quantum Electron.*, **26**, 8301312 (2020).

5. Sánchez, E., López-Hernández, A. & Pérez-López, D. Simulation of Highly Coupled Programmable Photonic Circuits. *J. Lightwave Technol*. **40**, 6423-6434 (2022).

6. Pérez-López, D. et al. Multipurpose silicon photonics signal processor core. *Nat. Commun*. **8**, 636 (2017).

7. Pérez-López, D. Gasulla, I., Capmany J. & Soref, R.A. Reconfigurable lattice mesh designs for programmable photonic processors. *Opt. Express* **24**, 12093-12106 (2016).

8. Marpaung, D., Morrison, B., Pant, R., Roeloffzen, C., Leinse, A., Hoekman, M., Heideman, R. & Eggleton, B. J. Si_3N_4 ring resonator-based microwave photonic notch filter with an ultrahigh peak rejection. *Opt. Express* **21** 23286–23294 (2013).

9. Liu, Y., Choudhary, A., Marpaung, D. & Eggleton, B.J. Integrated microwave photonic filters, *Adv. Opt. Photon.* **12**, 485–555 (2020).

10. Daulay, O., Liu,G., Guo, X., Eijkel, M. & Marpaung., D. A Tutorial on Integrated Microwave Photonics Spectral Shaping. *Journal of Lightw. Technology* **39**, 700-711, (2021).

11. Sánchez, E., Pérez-López, D., DasMahapatra, P. & Capmany, J. Modelling amplified arbitrary filtered microwave photonic links and systems. *Opt. Express* **29**, 14757-14772 (2021).

12. Pérez-López, D., Gasulla, I., Capmany, J., Fandiño, J., Muñoz, P. & Alavi, H. Figures of merit for self-beating filtered microwave photonic systems. *Opt. Express* **24**, 10087–10102 (2016).

13. Tripathi, S., Sabu, N. V., Gupta, A. K. & Dhillon, H. S. Millimeter wave and terahertz spectrum for 6G wireless, *in 6G Mobile Wireless Networks*. Springer, 83–121 (2021).

14. https://www.3gpp.org/DynaReport/38-series.htm.

15. Hasan M. & Hall., T. Rebuttal to “photonic microwave generation with frequency octupling based on a DP-QPSK modulator. *IEEE Photon. Technol. Lett.*, **28**, 1794–1794 (2016).

16. Zhang, H., Dong, Z., Wu, X. & Zhang, K. Generation of frequency eight fold millimeter-wave with optical carrier suppression by using one single-drive modulator. *Proc. Int. Symp. Intell. Signal Process. Commun. Syst.*, 353–356 (2017).

17. Malacarne, A., Bigongiari, A., D'Errico, A., Bogoni, A. & Porzi, C. Reconfigurable Low Phase Noise RF Carrier Generation up to W-Band in Silicon Photonics Technology. *Journal of Lightw. Technology*, **40**, 6891-6900 (2022).

18. Browning, C. et al. Gain-switched optical frequency combs for future mobile radio-over-fiber millimeter-wave systems. *Journal of Lightw. Technolgy* **36**, 4602–4610 (2018).

19. Wang, J., Shen, H., Fan, L. et al. Reconfigurable radio-frequency arbitrary waveforms synthesized in a silicon photonic chip. *Nat. Commun.* **6**, 5957 (2015).

20. Meijerink, A., Roeloffzen, C. G., Meijerink, R., Zhuang, L., Marpaung, D. A., Bentum, M. J., Burla, M., Verpoorte, J., Jorna, P., Hulzinga, A. et al. Novel ring resonator-based integrated photonic beam-former for broadband phased array receive antennas—part I: Design and performance analysis. *Journal of Lightw, Technology*, **28**, pp. 3–18, (2009).

21. Lenz, G., Eggleton, B., Madsen, C. K. & Slusher, R. Optical delay lines based on optical filters. *IEEE Journal of Quantum Electron.* **37** 525–532 (2001).

22. Xiang, C., Davenport, M. L., Khurgin, J. B., Morton, P. A. & Bowers, J. E. Low-loss continuously tunable optical true time delay based on Si_3N_4 ring resonators. *IEEE J. Sel. Top. Quantum Electron.* **24**, 1–9 (2017).

23. Zhu, C., Lu, L., Shan, W., Xu, W., Zhou, G., Zhou, L. & Chen, J. Silicon integrated microwave photonic beamformer. *Optica* **7** 1162–1170 (2020).

24. Waterhouse, R. & Novack, D. Realizing 5G: Microwave photonics for 5G mobile wireless systems. *IEEE Microwave Magazine* **16** 84–92 (2015).

25. Li, X., Yu, J. & Chang, G. K. Photonics-assisted technologies for extreme broadband 5G wireless communications. *J. Lightw. Technol.*, **37** 2851-2865 (2019).

26. Muniz, A. et al. Ultra-broadband photonics-based RF front-end toward 5G networks. *Journal of Optical Communications and Networking*. **8** B35-B42 (2016).

27. Serafino, G. et al. Photonics-assisted beamforming for 5G communications. *IEEE Photonics Technology Letters* **30** 1826-1829 (2018).

28. Schmogrow, R. et al. Error Vector Magnitude as a Performance Measure for Advanced Modulation Formats. *IEEE Photon. Technol. Lett.* **24** 61–63 (2012).

29. ETSI TS 138 521-1 V15.1.0 (2019-04) User Equipment (UE) conformance specification; Radio transmission and reception.

30. Chang, M. P., Blow, E. C., Lu, M. Z., Sun, J. J. & Prucnal, P. R. Integrated microwave photonic circuit for self-interference cancellation. *IEEE Trans. Microw. Theory Tech.* **65**, 4493–4501 (2017).

31. Yao, X. S. & Maleki, L. Converting light into spectrally pure microwave oscillation. *Opt. Lett.* **21**, 483–485, (1996).

32. Yao X. S. & Maleki, L. Multiloop optoelectronic oscillator. *IEEE J Quantum. Electron.* **36**, 79-84, (2000).
